# Supplementary material for: Viral Simulation Reveals Overestimation Bias in Within-Host Phylodynamic Migration Rate Estimates Under Selection
Source: Mol Biol Evol. 2026 Feb 2;43(2):msag014. doi: 10.1093/molbev/msag014 (PMC12911929; doi:10.1093/molbev/msag014)
Supplement: msag014_Supplementary_Data [file msag014_supplementary_data.pdf]

method	tree	population size	n samples	migration rate / mutation rate bp^{-1} generation^{-1}	0e+00	1e-03	2e-03	3e-03	4e-03	5e-03	6e-03	7e-03	8e-03	9e-03	1e-02
DTA	reconstructed	100	200	2.16e-5	0.60	0.60	0.57	0.56	0.62	0.54	0.57	0.53	0.55	0.52	0.58
				2.16e-4	0.60	0.62	0.53	0.57	0.54	0.50	0.48	0.48	0.38	0.46	0.42
			100	2.16e-5	0.58	0.57	0.55	0.59	0.64	0.49	0.58	0.52	0.51	0.52	0.56
				2.16e-4	0.59	0.60	0.51	0.56	0.51	0.50	0.50	0.49	0.39	0.44	0.41
			50	2.16e-5	0.56	0.57	0.55	0.55	0.61	0.46	0.56	0.49	0.54	0.50	0.59
				2.16e-4	0.56	0.57	0.47	0.52	0.50	0.44	0.42	0.43	0.38	0.41	0.36
		1000	200	2.16e-5	0.52	0.42	0.41	0.40	0.35	0.35	0.41	0.36	0.36	0.35	0.33
				2.16e-4	0.58	0.37	0.35	0.25	0.17	0.21	0.20	0.12	0.16	0.10	0.12
	true	100	200	2.16e-5	0.52	0.55	0.54	0.49	0.53	0.48	0.53	0.47	0.54	0.44	0.50
				2.16e-4	0.56	0.61	0.53	0.56	0.53	0.51	0.50	0.50	0.41	0.48	0.44
			100	2.16e-5	0.52	0.56	0.54	0.54	0.55	0.51	0.51	0.49	0.50	0.40	0.55
				2.16e-4	0.56	0.59	0.54	0.53	0.51	0.51	0.49	0.51	0.44	0.43	0.42
			50	2.16e-5	0.52	0.55	0.48	0.55	0.51	0.48	0.51	0.43	0.41	0.39	0.49
				2.16e-4	0.55	0.58	0.49	0.47	0.51	0.46	0.45	0.48	0.40	0.39	0.38
		1000	200	2.16e-5	0.49	0.35	0.40	0.34	0.37	0.37	0.36	0.27	0.29	0.24	0.20
				2.16e-4	0.48	0.31	0.30	0.22	0.15	0.20	0.17	0.11	0.15	0.11	0.16
MASCOT	reconstructed	100	200	2.16e-5	0.72	0.72	0.64	0.65	0.70	0.58	0.57	0.64	0.58	0.53	0.61
				2.16e-4	0.64	0.70	0.65	0.65	0.63	0.57	0.55	0.50	0.43	0.51	0.45
			100	2.16e-5	0.72	0.70	0.65	0.64	0.68	0.56	0.56	0.55	0.58	0.51	0.58
				2.16e-4	0.63	0.69	0.62	0.65	0.60	0.56	0.53	0.55	0.46	0.45	0.47
			50	2.16e-5	0.71	0.70	0.63	0.60	0.73	0.58	0.59	0.51	0.54	0.52	0.60
				2.16e-4	0.57	0.68	0.60	0.60	0.59	0.53	0.49	0.48	0.43	0.45	0.39
		1000	200	2.16e-5	0.52	0.44	0.43	0.42	0.37	0.35	0.41	0.36	0.36	0.34	0.32
				2.16e-4	0.60	0.37	0.37	0.27	0.19	0.22	0.21	0.13	0.17	0.11	0.13
	true	100	200	2.16e-5	0.72	0.72	0.63	0.61	0.66	0.58	0.54	0.59	0.59	0.48	0.57
				2.16e-4	0.64	0.70	0.66	0.64	0.62	0.57	0.56	0.52	0.44	0.52	0.47
			100	2.16e-5	0.71	0.67	0.63	0.57	0.57	0.52	0.44	0.47	0.58	0.51	0.52
				2.16e-4	0.61	0.68	0.60	0.57	0.54	0.50	0.50	0.51	0.37	0.44	0.48
			50	2.16e-5	0.69	0.64	0.60	0.57	0.57	0.51	0.50	0.44	0.42	0.49	0.46
				2.16e-4	0.56	0.62	0.53	0.50	0.51	0.48	0.50	0.46	0.38	0.37	0.38
		1000	200	2.16e-5	0.50	0.30	0.38	0.32	0.31	0.27	0.33	0.24	0.22	0.18	0.18
				2.16e-4	0.54	0.31	0.29	0.22	0.14	0.19	0.17	0.11	0.13	0.10	0.10

Table S1. Overestimation of Migration Rates from Selection with Strict Clock Model. This table shows the average posterior probability of overestimation for each migration rate corrected for the neutral baseline when using a strict clock during inference. The average is calculated over 50 paired simulations where the posterior probability of overestimation is derived from 1000 migration rate samples. The values are colored according to the posterior probability, with red indicating a high probability of overestimation and blue indicating a high probability of underestimation.

				selection											neutral											lognormal
method	tree	population size	n samples	migration rate / mutation rate bp^{-1} generation^{-1}	0e+00	1e-03	2e-03	3e-03	4e-03	5e-03	6e-03	7e-03	8e-03	9e-03	1e-02	0e+00	1e-03	2e-03	3e-03	4e-03	5e-03	6e-03	7e-03	8e-03	9e-03	1e-02
DTA	reconstructed	100	200	2.16e-5	1.00	0.68	0.60	0.58	0.58	0.53	0.55	0.56	0.53	0.56	0.51	1.00	0.85	0.77	0.72	0.79	0.70	0.73	0.72	0.70	0.67	0.70
				2.16e-4	1.00	0.71	0.63	0.59	0.61	0.61	0.57	0.56	0.66	0.54	0.59	1.00	0.93	0.85	0.88	0.88	0.83	0.85	0.81	0.80	0.82	0.80
			100	2.16e-5	1.00	0.72	0.65	0.60	0.61	0.57	0.58	0.60	0.57	0.54	0.58	1.00	0.88	0.80	0.79	0.81	0.73	0.73	0.73	0.69	0.70	0.75
				2.16e-4	1.00	0.74	0.65	0.61	0.61	0.62	0.56	0.54	0.65	0.60	0.60	1.00	0.94	0.88	0.88	0.89	0.86	0.85	0.80	0.82	0.81	0.78
			50	2.16e-5	1.00	0.77	0.69	0.64	0.67	0.61	0.60	0.66	0.62	0.67	0.62	1.00	0.92	0.87	0.84	0.86	0.78	0.77	0.80	0.74	0.74	0.77
				2.16e-4	1.00	0.76	0.72	0.65	0.67	0.67	0.64	0.60	0.70	0.67	0.65	1.00	0.95	0.90	0.90	0.90	0.85	0.88	0.82	0.83	0.82	0.84
		1000	200	2.16e-5	1.00	0.60	0.51	0.43	0.39	0.38	0.33	0.35	0.46	0.37	0.46	1.00	0.84	0.82	0.83	0.75	0.74	0.78	0.77	0.80	0.74	0.74
				2.16e-4	1.00	0.54	0.39	0.42	0.42	0.37	0.34	0.43	0.41	0.39	0.42	1.00	0.87	0.87	0.87	0.85	0.91	0.86	0.88	0.88	0.86	0.90
	true	100	200	2.16e-5	1.00	0.72	0.66	0.65	0.67	0.64	0.64	0.65	0.60	0.69	0.63	1.00	0.79	0.73	0.66	0.72	0.64	0.70	0.65	0.68	0.62	0.63
				2.16e-4	1.00	0.74	0.69	0.65	0.69	0.67	0.64	0.63	0.72	0.63	0.65	1.00	0.88	0.77	0.79	0.78	0.73	0.73	0.69	0.67	0.68	0.65
			100	2.16e-5	1.00	0.52	0.34	0.34	0.27	0.25	0.22	0.27	0.24	0.23	0.19	1.00	0.60	0.33	0.26	0.35	0.21	0.15	0.21	0.19	0.11	0.16
				2.16e-4	1.00	0.61	0.54	0.49	0.47	0.39	0.41	0.35	0.43	0.36	0.41	1.00	0.85	0.67	0.59	0.61	0.56	0.62	0.50	0.50	0.50	0.43
			50	2.16e-5	1.00	0.59	0.40	0.48	0.40	0.31	0.29	0.34	0.35	0.35	0.29	1.00	0.60	0.41	0.40	0.47	0.33	0.35	0.38	0.30	0.27	0.26
				2.16e-4	1.00	0.55	0.56	0.44	0.41	0.41	0.34	0.36	0.43	0.37	0.43	1.00	0.81	0.67	0.65	0.59	0.55	0.54	0.47	0.47	0.47	0.43
		1000	200	2.16e-5	1.00	0.14	0.10	0.08	0.05	0.06	0.04	0.05	0.08	0.10	0.10	1.00	0.21	0.16	0.09	0.05	0.05	0.04	0.04	0.04	0.01	0.06
				2.16e-4	1.00	0.15	0.09	0.05	0.04	0.05	0.03	0.06	0.05	0.05	0.08	1.00	0.41	0.26	0.31	0.17	0.21	0.18	0.14	0.16	0.13	0.13
MASCOT	reconstructed	100	200	2.16e-5	1.00	0.64	0.56	0.56	0.55	0.50	0.53	0.53	0.50	0.54	0.47	1.00	0.85	0.78	0.73	0.80	0.71	0.73	0.72	0.70	0.67	0.70
				2.16e-4	1.00	0.71	0.61	0.58	0.60	0.60	0.55	0.56	0.64	0.52	0.57	1.00	0.93	0.85	0.88	0.88	0.83	0.85	0.81	0.81	0.82	0.81
			100	2.16e-5	1.00	0.65	0.60	0.59	0.56	0.55	0.54	0.58	0.48	0.56	0.55	1.00	0.89	0.80	0.79	0.82	0.71	0.75	0.75	0.72	0.72	0.75
				2.16e-4	1.00	0.72	0.62	0.59	0.62	0.58	0.61	0.56	0.64	0.55	0.60	1.00	0.95	0.89	0.87	0.88	0.85	0.87	0.80	0.83	0.80	0.80
			50	2.16e-5	1.00	0.71	0.63	0.62	0.63	0.57	0.59	0.57	0.57	0.64	0.57	1.00	0.92	0.88	0.83	0.87	0.81	0.79	0.80	0.75	0.76	0.81
				2.16e-4	1.00	0.73	0.67	0.63	0.58	0.65	0.63	0.54	0.65	0.56	0.65	1.00	0.96	0.91	0.88	0.89	0.87	0.88	0.84	0.84	0.84	0.80
		1000	200	2.16e-5	1.00	0.60	0.50	0.42	0.39	0.37	0.32	0.34	0.44	0.36	0.45	1.00	0.85	0.82	0.84	0.75	0.75	0.79	0.78	0.81	0.75	0.74
				2.16e-4	1.00	0.54	0.39	0.41	0.41	0.36	0.33	0.42	0.41	0.38	0.41	1.00	0.87	0.87	0.87	0.85	0.91	0.86	0.88	0.88	0.87	0.90
	true	100	200	2.16e-5	1.00	0.61	0.53	0.54	0.53	0.50	0.50	0.50	0.42	0.52	0.43	1.00	0.82	0.76	0.66	0.71	0.63	0.66	0.61	0.64	0.56	0.59
				2.16e-4	1.00	0.70	0.60	0.58	0.59	0.59	0.54	0.56	0.63	0.50	0.56	1.00	0.93	0.85	0.87	0.88	0.83	0.84	0.81	0.80	0.82	0.80
			100	2.16e-5	1.00	0.68	0.69	0.67	0.69	0.72	0.71	0.68	0.63	0.72	0.65	1.00	0.90	0.85	0.82	0.84	0.77	0.78	0.80	0.77	0.75	0.75
				2.16e-4	1.00	0.75	0.67	0.67	0.67	0.68	0.68	0.67	0.68	0.64	0.65	1.00	0.94	0.89	0.89	0.90	0.87	0.87	0.83	0.87	0.85	0.80
			50	2.16e-5	1.00	0.73	0.68	0.71	0.72	0.72	0.76	0.75	0.70	0.73	0.70	1.00	0.93	0.88	0.81	0.90	0.82	0.80	0.84	0.80	0.77	0.78
				2.16e-4	1.00	0.75	0.69	0.72	0.72	0.73	0.69	0.71	0.69	0.75	0.76	1.00	0.96	0.91	0.90	0.90	0.90	0.87	0.88	0.90	0.85	0.86
		1000	200	2.16e-5	1.00	0.78	0.68	0.68	0.64	0.65	0.66	0.73	0.74	0.72	0.82	1.00	0.85	0.87	0.86	0.85	0.85	0.89	0.86	0.88	0.81	0.75
				2.16e-4	1.00	0.62	0.57	0.56	0.60	0.52	0.55	0.66	0.68	0.68	0.71	1.00	0.88	0.89	0.89	0.88	0.93	0.90	0.92	0.93	0.92	0.94

Table S2. Overview of Posterior Overestimation Probability of Migration Rates with Relaxed Clock Model. This table shows the average posterior probability of overestimation for each migration rate when using a strict clock during inference. The posterior probability of overestimation is derived from 1000 migration rate samples. The values are colored according to the posterior probability, with red indicating a high probability of overestimation and blue indicating a high probability of underestimation.

				selection											neutral											lognormal
method	tree	population size	n samples	migration rate / mutation rate bp^{-1} generation^{-1}	0e+00	1e-03	2e-03	3e-03	4e-03	5e-03	6e-03	7e-03	8e-03	9e-03	1e-02	0e+00	1e-03	2e-03	3e-03	4e-03	5e-03	6e-03	7e-03	8e-03	9e-03	1e-02
DTA	reconstructed	100	200	2.16e-5	1.00	0.62	0.53	0.51	0.50	0.46	0.48	0.50	0.46	0.49	0.45	1.00	0.74	0.63	0.59	0.66	0.54	0.57	0.54	0.54	0.52	0.55
				2.16e-4	1.00	0.58	0.49	0.43	0.46	0.43	0.42	0.37	0.47	0.37	0.41	1.00	0.75	0.54	0.52	0.52	0.42	0.40	0.33	0.32	0.31	0.29
			100	2.16e-5	1.00	0.70	0.60	0.51	0.52	0.55	0.48	0.51	0.54	0.54	0.51	1.00	0.78	0.67	0.64	0.70	0.54	0.60	0.55	0.54	0.56	0.59
				2.16e-4	1.00	0.65	0.58	0.48	0.55	0.48	0.48	0.41	0.55	0.46	0.48	1.00	0.79	0.61	0.57	0.55	0.48	0.50	0.39	0.39	0.37	0.35
			50	2.16e-5	1.00	0.73	0.67	0.63	0.55	0.62	0.57	0.63	0.57	0.63	0.56	1.00	0.81	0.72	0.69	0.72	0.56	0.62	0.62	0.62	0.61	0.66
				2.16e-4	1.00	0.71	0.69	0.57	0.58	0.58	0.62	0.56	0.65	0.56	0.61	1.00	0.81	0.67	0.62	0.57	0.51	0.55	0.46	0.50	0.46	0.40
		1000	200	2.16e-5	1.00	0.40	0.35	0.30	0.25	0.25	0.20	0.22	0.33	0.27	0.35	1.00	0.31	0.24	0.19	0.13	0.15	0.15	0.15	0.19	0.14	0.19
				2.16e-4	1.00	0.33	0.20	0.23	0.23	0.20	0.19	0.25	0.26	0.23	0.27	1.00	0.20	0.08	0.05	0.01	0.02	0.01	0.01	0.01	0.01	0.00
	true	100	200	2.16e-5	1.00	0.71	0.65	0.64	0.64	0.62	0.62	0.64	0.58	0.66	0.60	1.00	0.77	0.71	0.62	0.69	0.60	0.65	0.61	0.63	0.57	0.59
				2.16e-4	1.00	0.73	0.68	0.63	0.67	0.65	0.63	0.61	0.70	0.60	0.62	1.00	0.85	0.72	0.72	0.73	0.65	0.65	0.60	0.57	0.57	0.55
			100	2.16e-5	1.00	0.84	0.77	0.74	0.74	0.72	0.70	0.71	0.69	0.74	0.63	1.00	0.88	0.79	0.79	0.81	0.74	0.73	0.69	0.69	0.60	0.69
				2.16e-4	1.00	0.80	0.71	0.69	0.71	0.65	0.67	0.58	0.68	0.64	0.63	1.00	0.88	0.76	0.74	0.73	0.67	0.67	0.61	0.59	0.57	0.54
			50	2.16e-5	1.00	0.85	0.83	0.73	0.73	0.70	0.74	0.78	0.74	0.77	0.68	1.00	0.89	0.81	0.80	0.77	0.69	0.78	0.72	0.65	0.63	0.67
				2.16e-4	1.00	0.85	0.80	0.76	0.71	0.72	0.74	0.64	0.75	0.73	0.72	1.00	0.90	0.79	0.77	0.74	0.68	0.69	0.61	0.64	0.59	0.59
		1000	200	2.16e-5	1.00	0.77	0.70	0.66	0.59	0.57	0.55	0.62	0.62	0.60	0.68	1.00	0.58	0.57	0.44	0.38	0.39	0.35	0.28	0.32	0.22	0.21
				2.16e-4	1.00	0.64	0.51	0.57	0.57	0.50	0.50	0.62	0.62	0.55	0.61	1.00	0.38	0.24	0.20	0.09	0.12	0.09	0.08	0.07	0.05	0.07
MASCOT	reconstructed	100	200	2.16e-5	1.00	0.41	0.41	0.37	0.35	0.38	0.42	0.38	0.38	0.45	0.39	1.00	0.72	0.63	0.56	0.63	0.51	0.51	0.57	0.50	0.47	0.55
				2.16e-4	1.00	0.44	0.32	0.31	0.35	0.34	0.33	0.32	0.41	0.31	0.37	1.00	0.74	0.54	0.51	0.52	0.40	0.39	0.32	0.31	0.29	0.28
			100	2.16e-5	1.00	0.47	0.42	0.43	0.39	0.42	0.46	0.46	0.42	0.52	0.47	1.00	0.76	0.64	0.62	0.67	0.50	0.54	0.53	0.53	0.52	0.57
				2.16e-4	1.00	0.50	0.41	0.37	0.42	0.38	0.46	0.33	0.45	0.41	0.41	1.00	0.78	0.59	0.58	0.57	0.46	0.47	0.40	0.39	0.33	0.34
			50	2.16e-5	1.00	0.51	0.51	0.51	0.42	0.45	0.49	0.59	0.50	0.60	0.50	1.00	0.79	0.70	0.65	0.76	0.56	0.59	0.61	0.53	0.60	0.60
				2.16e-4	1.00	0.55	0.51	0.46	0.48	0.52	0.53	0.45	0.53	0.48	0.55	1.00	0.80	0.67	0.62	0.63	0.55	0.53	0.41	0.45	0.40	0.40
		1000	200	2.16e-5	1.00	0.37	0.32	0.27	0.23	0.23	0.19	0.20	0.31	0.25	0.33	1.00	0.31	0.24	0.19	0.12	0.14	0.14	0.13	0.17	0.12	0.17
				2.16e-4	1.00	0.31	0.18	0.20	0.21	0.19	0.17	0.23	0.24	0.21	0.25	1.00	0.20	0.08	0.05	0.01	0.02	0.01	0.01	0.01	0.01	0.00
	true	100	200	2.16e-5	1.00	0.37	0.38	0.36	0.35	0.37	0.39	0.35	0.32	0.44	0.36	1.00	0.68	0.58	0.50	0.52	0.46	0.42	0.46	0.45	0.41	0.46
				2.16e-4	1.00	0.44	0.32	0.32	0.36	0.35	0.33	0.33	0.40	0.29	0.36	1.00	0.73	0.55	0.51	0.53	0.42	0.40	0.34	0.32	0.30	0.30
			100	2.16e-5	1.00	0.57	0.62	0.56	0.57	0.63	0.67	0.64	0.56	0.64	0.60	1.00	0.79	0.77	0.66	0.71	0.63	0.61	0.61	0.68	0.60	0.61
				2.16e-4	1.00	0.61	0.54	0.51	0.55	0.56	0.56	0.54	0.63	0.56	0.52	1.00	0.83	0.70	0.64	0.64	0.57	0.55	0.54	0.45	0.47	0.49
			50	2.16e-5	1.00	0.65	0.64	0.62	0.63	0.66	0.69	0.71	0.75	0.65	0.71	1.00	0.83	0.79	0.73	0.76	0.69	0.71	0.63	0.65	0.65	0.64
				2.16e-4	1.00	0.72	0.66	0.66	0.63	0.65	0.65	0.62	0.71	0.66	0.63	1.00	0.87	0.74	0.70	0.67	0.65	0.67	0.59	0.58	0.50	0.50
		1000	200	2.16e-5	1.00	0.71	0.61	0.60	0.54	0.54	0.55	0.59	0.65	0.62	0.73	1.00	0.43	0.42	0.33	0.26	0.26	0.30	0.22	0.25	0.17	0.20
				2.16e-4	1.00	0.55	0.48	0.48	0.52	0.45	0.45	0.54	0.56	0.52	0.57	1.00	0.30	0.21	0.15	0.05	0.08	0.07	0.05	0.04	0.03	0.03

Table S3. Overview of Posterior Overestimation Probability of Migration Rates with Strict Clock Model. This table shows the average posterior probability of overestimation for each migration rate when using a relaxed clock during inference. The posterior probability of overestimation is derived from 1000 migration rate samples. The values are colored according to the posterior probability, with red indicating a high probability of overestimation and blue indicating a high probability of underestimation.

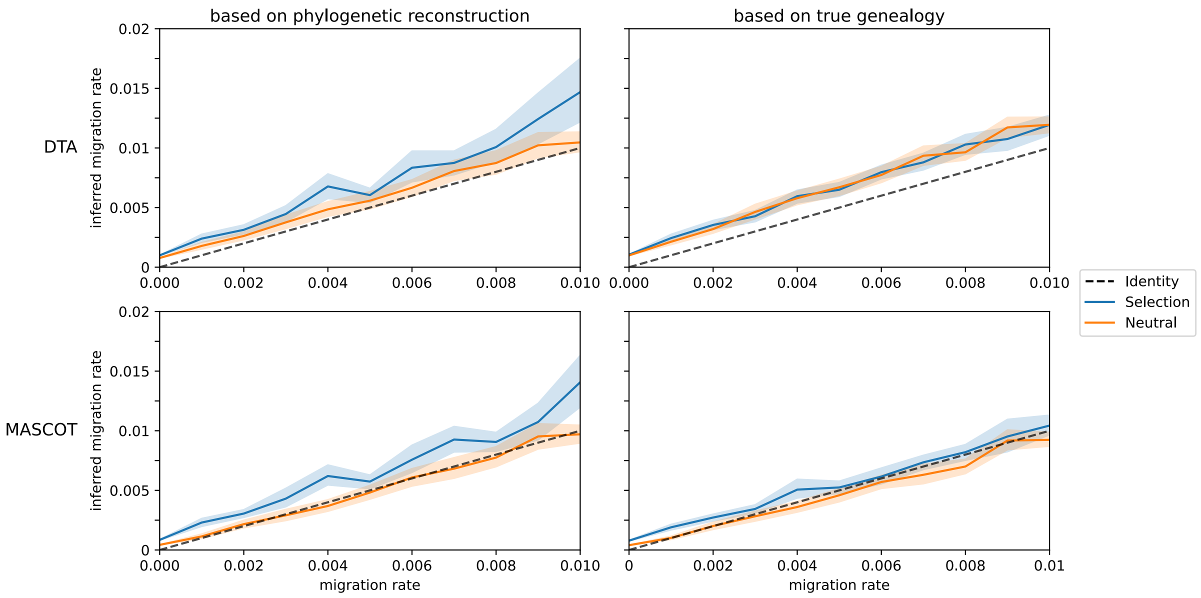

Fig. S1: **Strict Clock Model.** Average mean migration rate estimate with DTA and MASCOT in BEAST2. For each migration rate, 50 simulations were conducted, maintaining a constant population of 100 virions in each compartment, with a mutation rate of 2.16×10^{-5} mutation bp^{-1} generation^{-1}, and spanning a total of 1,000 generations. All sequences were sampled and analyzed in BEAST2 with a strict clock model. The left figure illustrates the inference while sampling trees, while the right figure displays the migration rate estimates based on the true–simulated–genealogy. The lighter colored bands illustrate the 95% confidence interval.

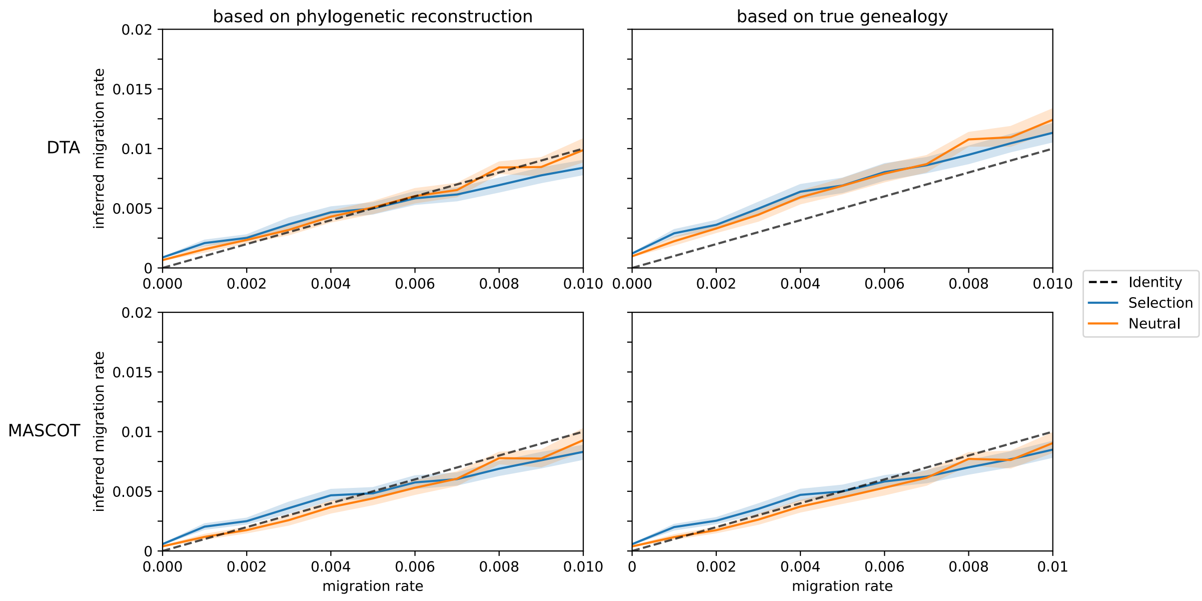

Fig. S2: **Strict Clock Model with Increased Mutation Rate.** Average mean migration rate estimate with DTA and MASCOT in BEAST2. For each migration rate, 50 simulations were conducted, maintaining a constant population of 100 virions in each compartment, with a mutation rate of 2.16×10^{-4} mutation bp^{-1} generation^{-1}, and spanning a total of 1,000 generations. All sequences were sampled and analyzed in BEAST2 with a strict clock model. The left figure illustrates the inference while sampling trees, while the right figure displays the migration rate estimates based on the true–simulated–genealogy. The lighter colored bands illustrate the 95% confidence interval.

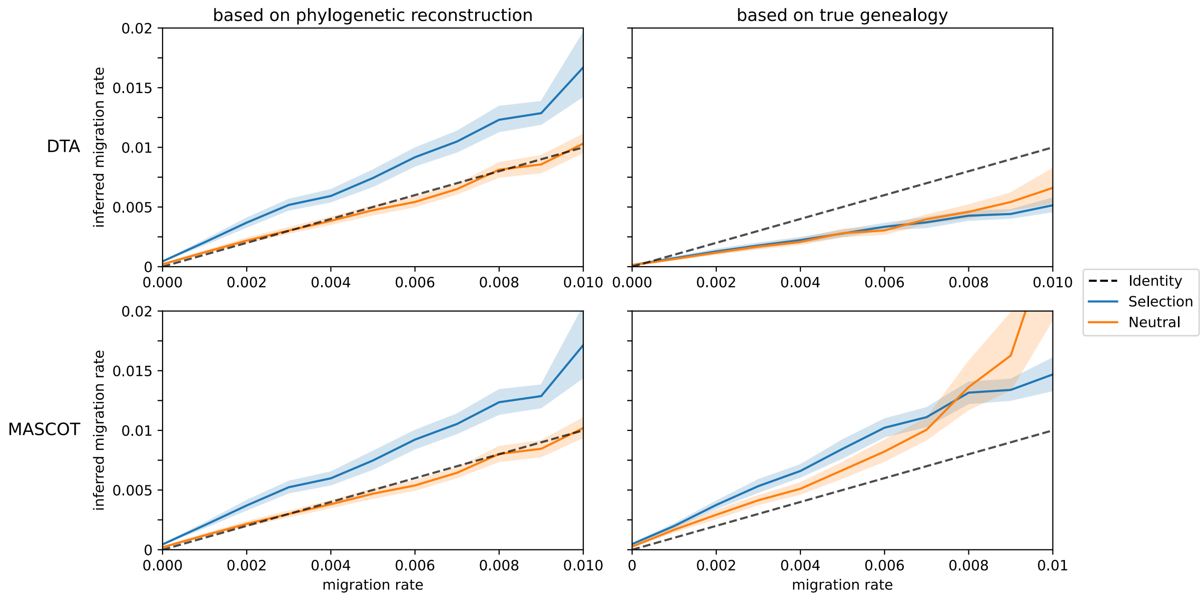

Fig. S3: **Loss of Accuracy in Migration Rate Estimates when Population Size Increases.** Average mean migration rate estimate with DTA and MASCOT in BEAST2. For each migration rate, 50 simulations were conducted, maintaining a constant population of 1000 virions in each compartment, with a mutation rate of 2.16×10^{-5} mutation bp^{-1} generation^{-1}, and spanning a total of 1,000 generations. All sequences were sampled and analyzed in BEAST2 with a relaxed clock model. The left figure illustrates the inference while sampling trees, while the right figure displays the migration rate estimates based on the true–simulated–genealogy. The lighter colored bands illustrate the 95% confidence interval.

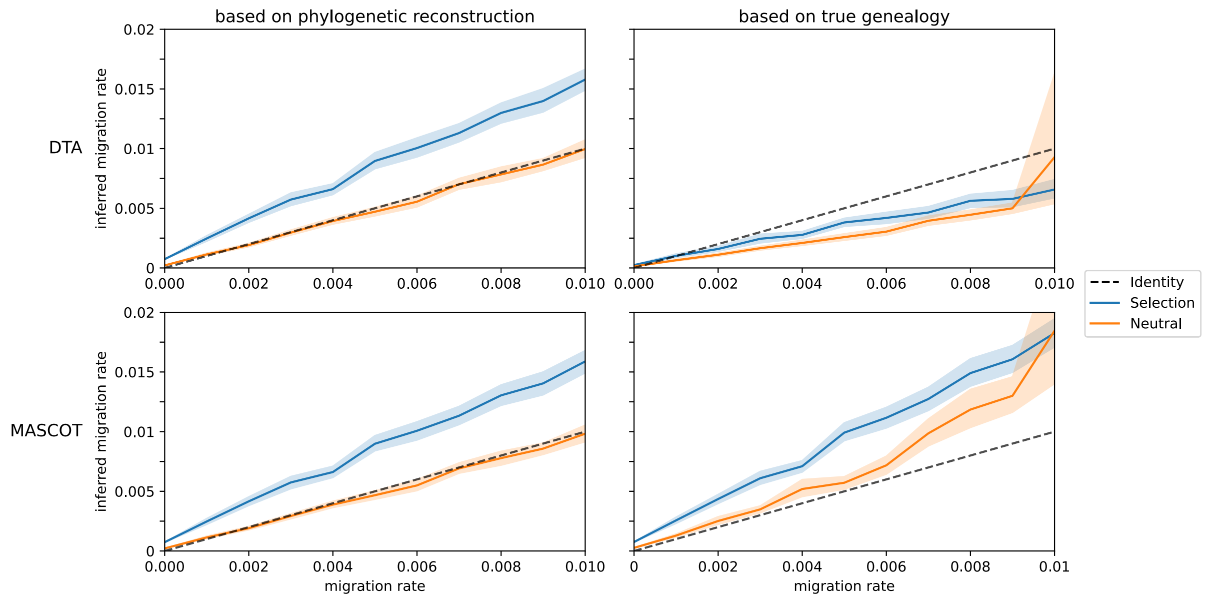

Fig. S4: **Increased Mutation Rate and Population Size with Relaxed Clock Model.** Average mean migration rate estimate with DTA and MASCOT in BEAST2. For each migration rate, 50 simulations were conducted, maintaining a constant population of 1000 virions in each compartment, with a mutation rate of 2.16×10^{-4} mutation bp^{-1} generation^{-1}, and spanning a total of 1,000 generations. All sequences were sampled and analyzed in BEAST2 with a relaxed clock model. The left figure illustrates the inference while sampling trees, while the right figure displays the migration rate estimates based on the true–simulated–genealogy. The lighter colored bands illustrate the 95% confidence interval.

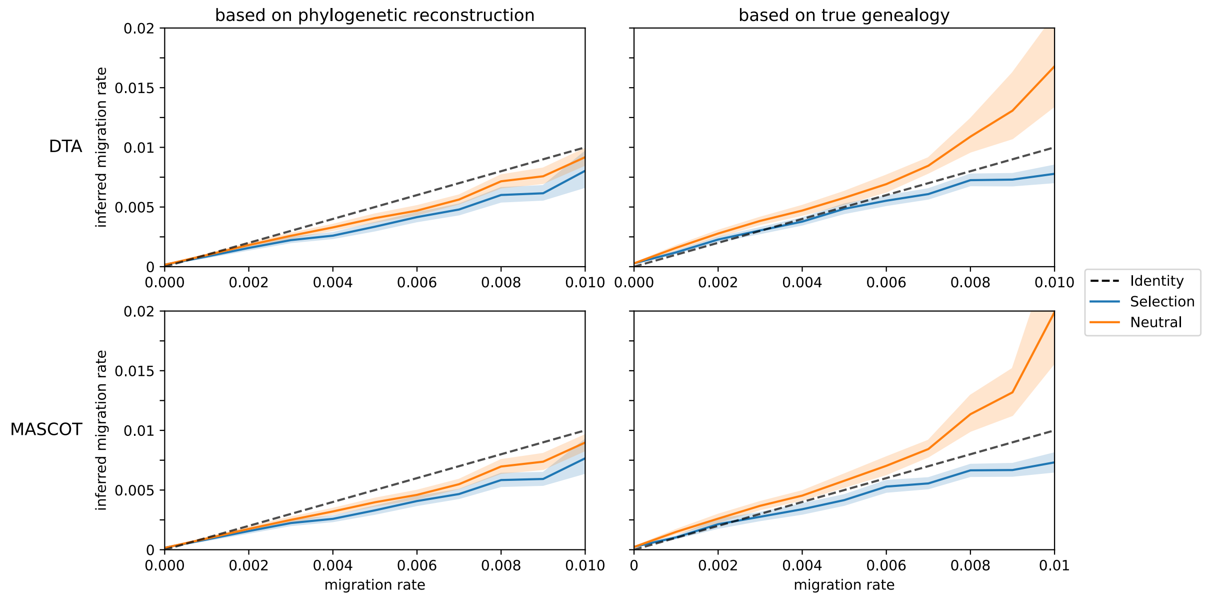

Fig. S5: **Increased Population Size with Strict Clock Model.** Average mean migration rate estimate with DTA and MASCOT in BEAST2. For each migration rate, 50 simulations were conducted, maintaining a constant population of 1000 virions in each compartment, with a mutation rate of 2.16×10^{-5} mutation bp^{-1} generation^{-1}, and spanning a total of 1,000 generations. All sequences were sampled and analyzed in BEAST2 with a strict clock model. The left figure illustrates the inference while sampling trees, while the right figure displays the migration rate estimates based on the true–simulated–genealogy. The lighter colored bands illustrate the 95% confidence interval.

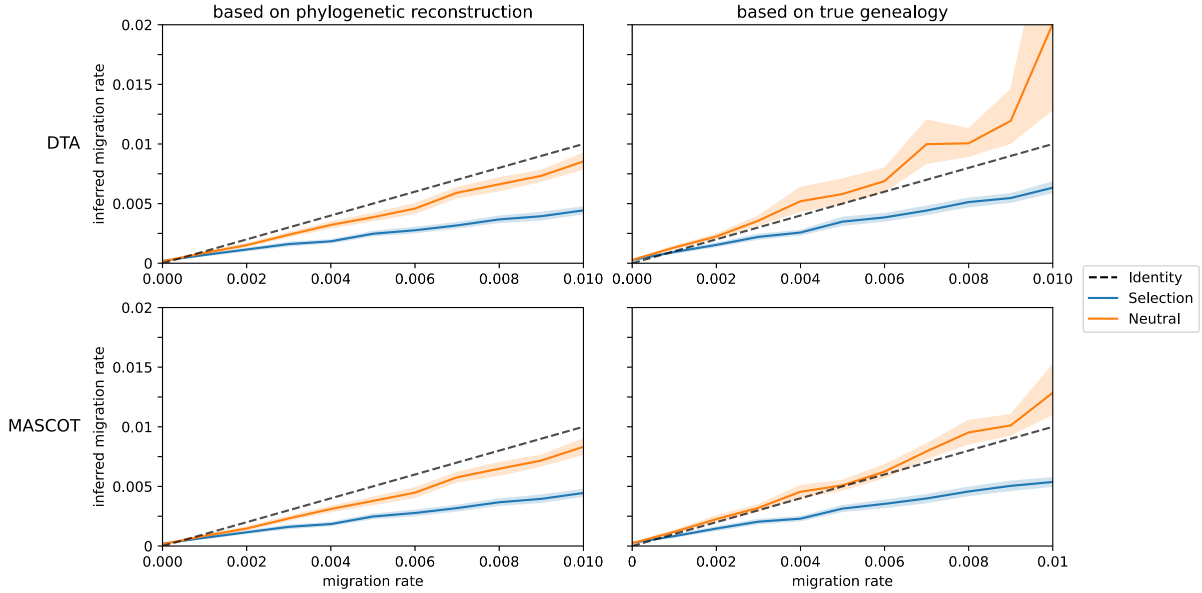

Fig. S6: **Increased Mutation Rate and Population Size with Strict Clock Model.** Average mean migration rate estimate with DTA and MASCOT in BEAST2. For each migration rate, 50 simulations were conducted, maintaining a constant population of 1000 virions in each compartment, with a mutation rate of 2.16×10^{-4} mutation bp^{-1} generation^{-1}, and spanning a total of 1,000 generations. All sequences were sampled and analyzed in BEAST2 with a strict clock model. The left figure illustrates the inference while sampling trees, while the right figure displays the migration rate estimates based on the true–simulated–genealogy. The lighter colored bands illustrate the 95% confidence interval.

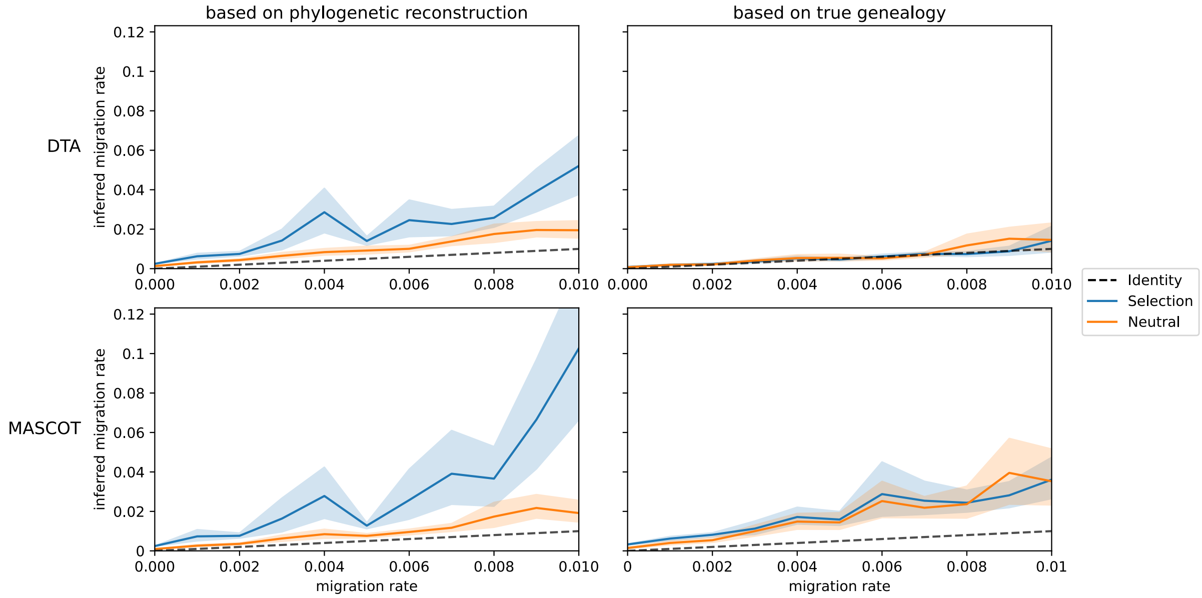

Fig. S7: **Amplified Bias caused by Sampling (relaxed clock).** Average mean migration rate estimate with DTA and MASCOT in BEAST2. For each migration rate, 50 simulations were conducted, maintaining a constant population of 100 virions in each compartment, with a mutation rate of 2.16×10^{-5} mutation bp^{-1} generation^{-1}, and spanning a total of 1,000 generations. All sequences were sampled and analyzed in BEAST2 with a relaxed clock model. The left figure illustrates the inference while sampling trees, while the right figure displays the migration rate estimates based on the true–simulated–genealogy. The lighter colored bands illustrate the 95% confidence interval. Here, inference was performed on 50 samples of simulated sequences.

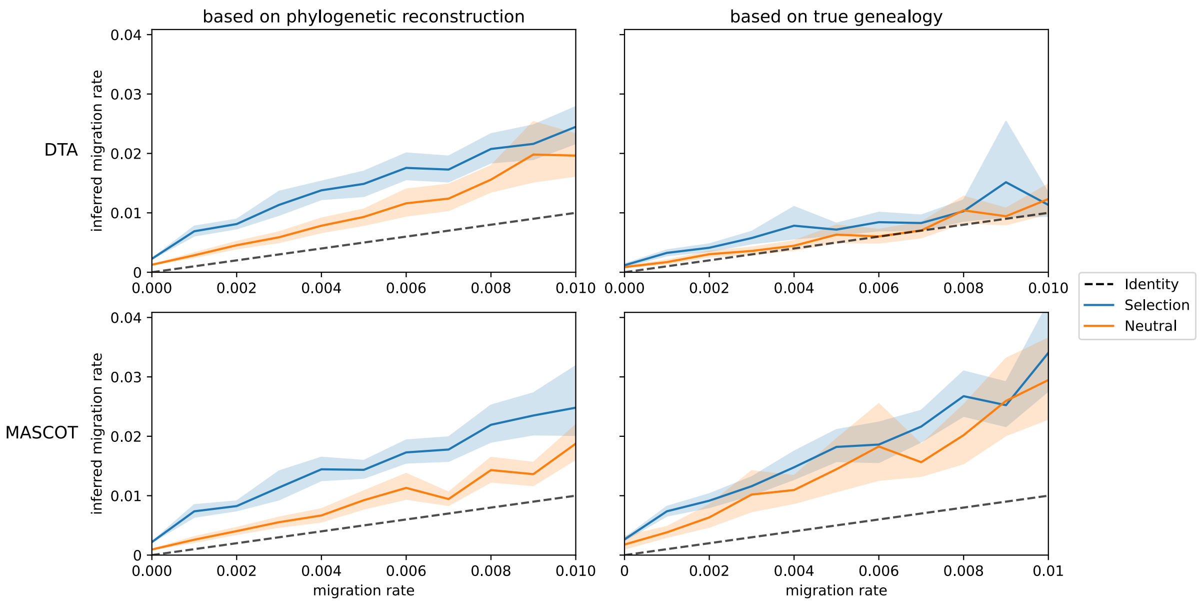

Fig. S8: **Amplified Bias caused by Sampling (relaxed clock, increased migration rate).** Average mean migration rate estimate with DTA and MASCOT in BEAST2. For each migration rate, 50 simulations were conducted, maintaining a constant population of 100 virions in each compartment, with a mutation rate of 2.16×10^{-4} mutation bp^{-1} generation^{-1}, and spanning a total of 1,000 generations. All sequences were sampled and analyzed in BEAST2 with a relaxed clock model. The left figure illustrates the inference while sampling trees, while the right figure displays the migration rate estimates based on the true–simulated–genealogy. The lighter colored bands illustrate the 95% confidence interval. Here, inference was performed on 50 samples of simulated sequences.

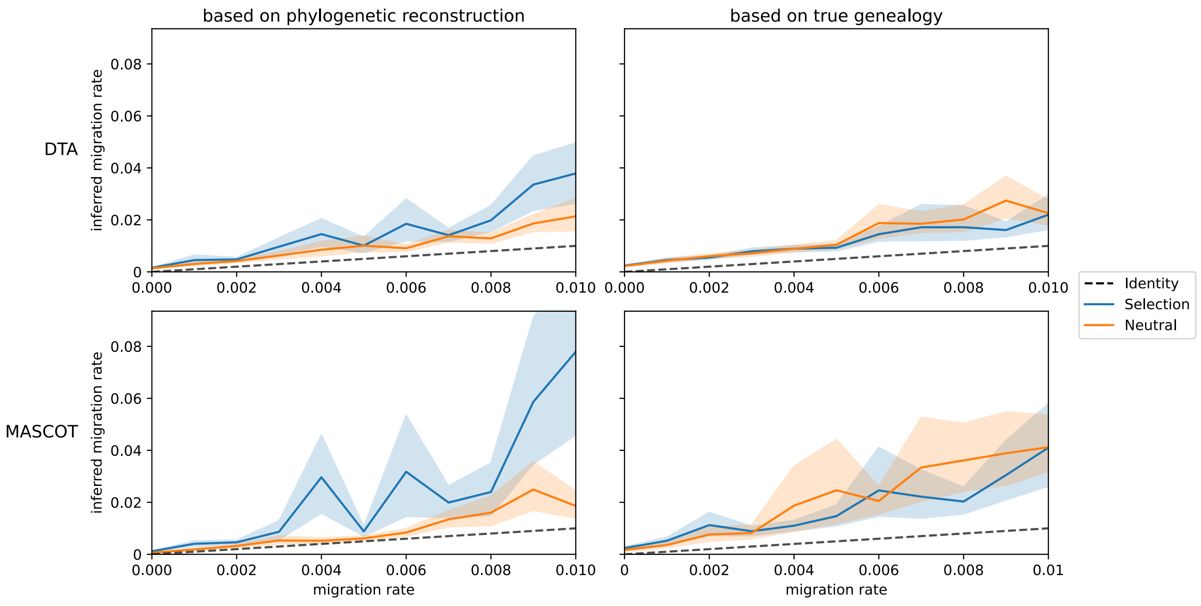

Fig. S9: **Amplified Bias caused by Sampling (strict clock).** Average mean migration rate estimate with DTA and MASCOT in BEAST2. For each migration rate, 50 simulations were conducted, maintaining a constant population of 100 virions in each compartment, with a mutation rate of 2.16×10^{-5} mutation bp^{-1} generation^{-1}, and spanning a total of 1,000 generations. All sequences were sampled and analyzed in BEAST2 with a strict clock model. The left figure illustrates the inference while sampling trees, while the right figure displays the migration rate estimates based on the true–simulated–genealogy. The lighter colored bands illustrate the 95% confidence interval. Here, inference was performed on 50 samples of simulated sequences.

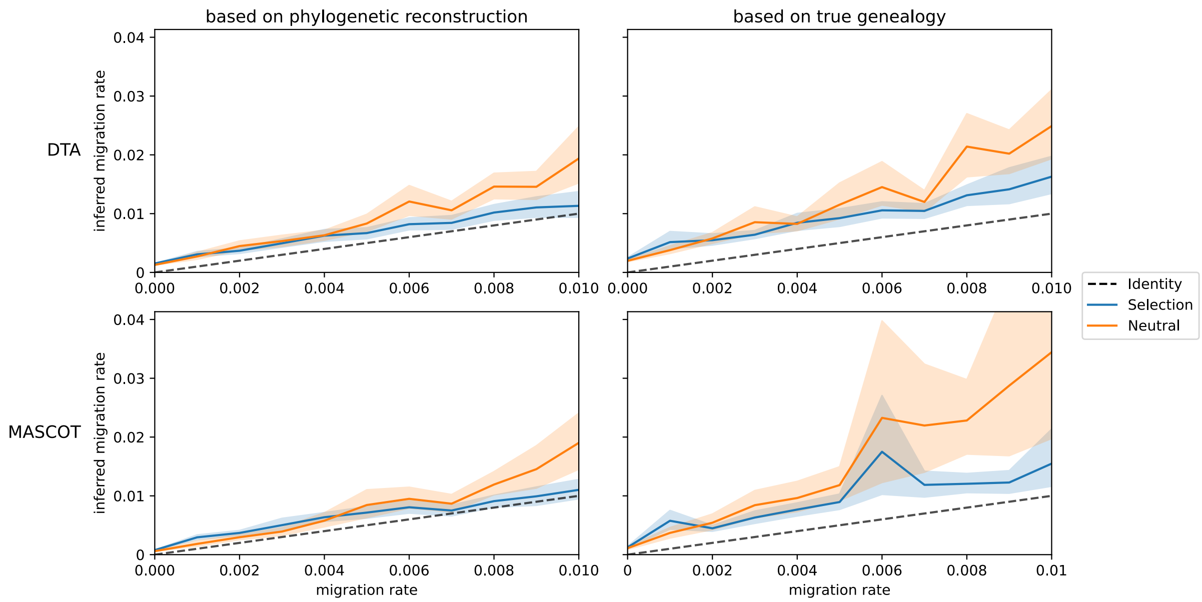

Fig. S10: **Amplified Bias caused by Sampling (strict clock, increased migration rate).** Average mean migration rate estimate with DTA and MASCOT in BEAST2. For each migration rate, 50 simulations were conducted, maintaining a constant population of 100 virions in each compartment, with a mutation rate of 2.16×10^{-4} mutation bp^{-1} generation^{-1}, and spanning a total of 1,000 generations. All sequences were sampled and analyzed in BEAST2 with a strict clock model. The left figure illustrates the inference while sampling trees, while the right figure displays the migration rate estimates based on the true–simulated–genealogy. The lighter colored bands illustrate the 95% confidence interval. Here, inference was performed on 50 samples of simulated sequences.

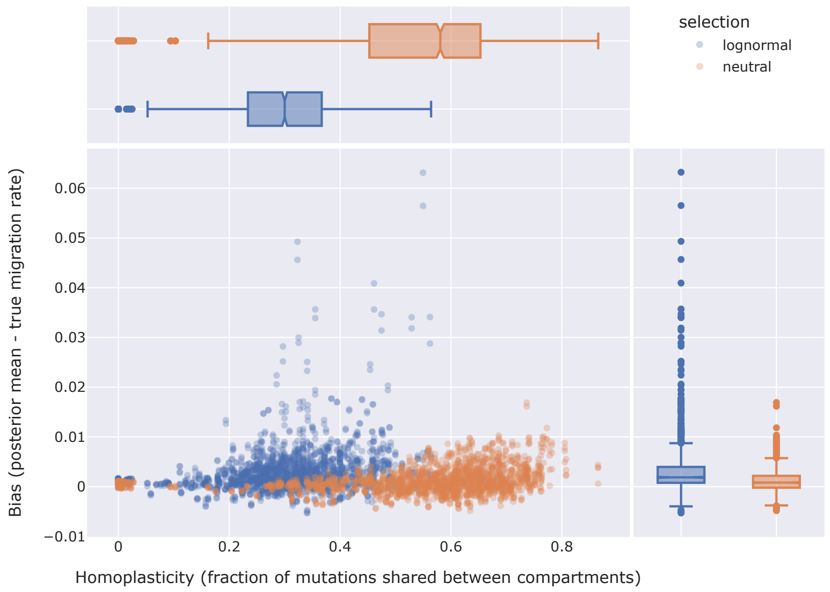

Fig. S11: Homoplasticity vs. Bias in migration rate estimates with relaxed clock model on simulations with a constant population size of 100 virions per compartment and a mutation rate of 2.16×10^{-5} mutation bp^{-1} generation^{-1}.

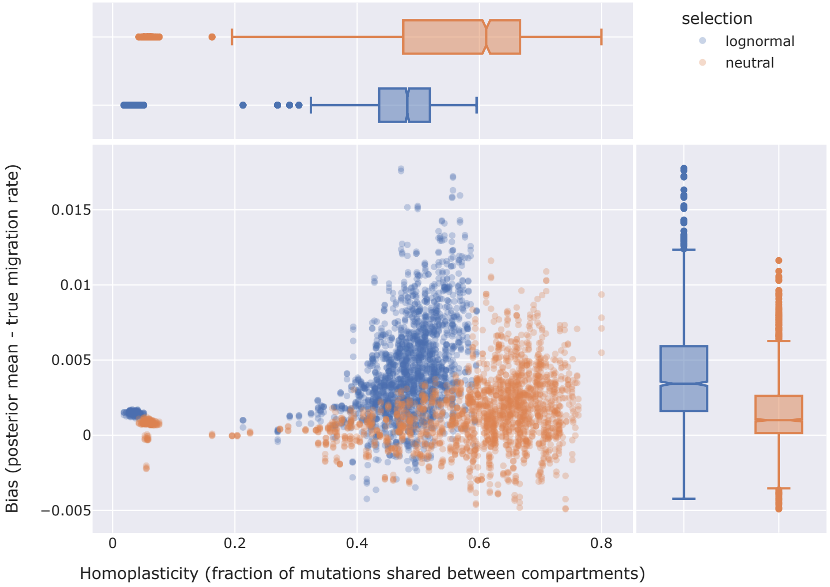

Fig. S12: Homoplasticity vs. Bias in migration rate estimates with relaxed clock model on simulations with a constant population size of 100 virions per compartment and a mutation rate of 2.16×10^{-4} mutation bp^{-1} generation^{-1}.

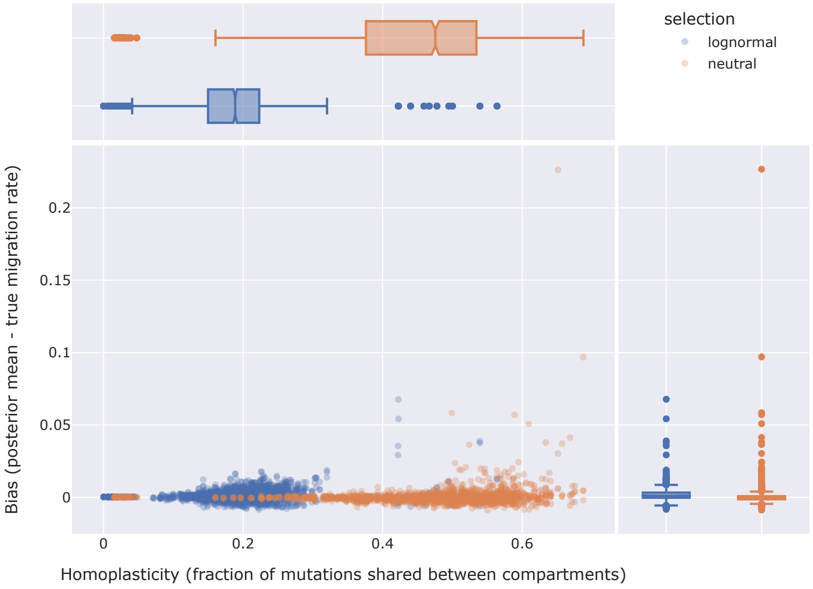

Fig. S13: Homoplasticity vs. Bias in migration rate estimates with relaxed clock model on simulations with a constant population size of 1000 virions per compartment and a mutation rate of 2.16×10^{-5} mutation bp^{-1} generation^{-1}.

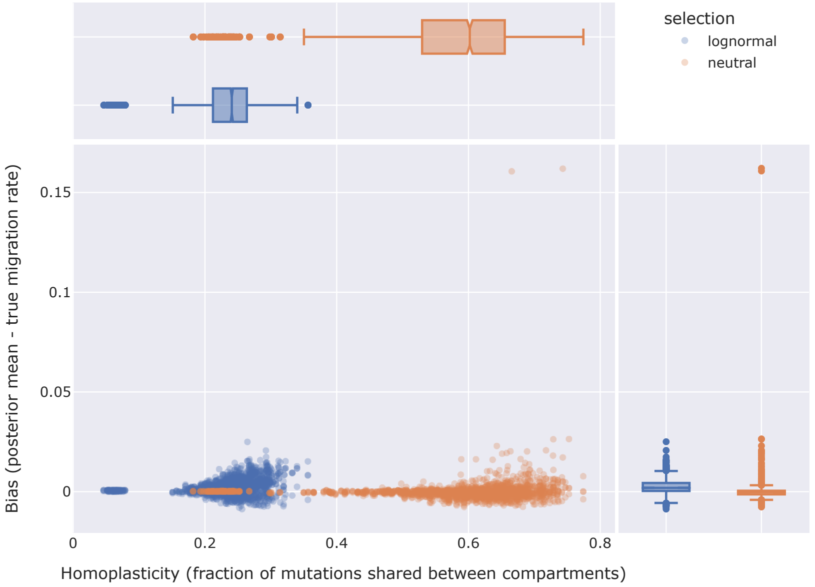

Fig. S14: Homoplasticity vs. Bias in migration rate estimates with relaxed clock model on simulations with a constant population size of 1000 virions per compartment and a mutation rate of 2.16×10^{-4} mutation bp^{-1} generation^{-1}.

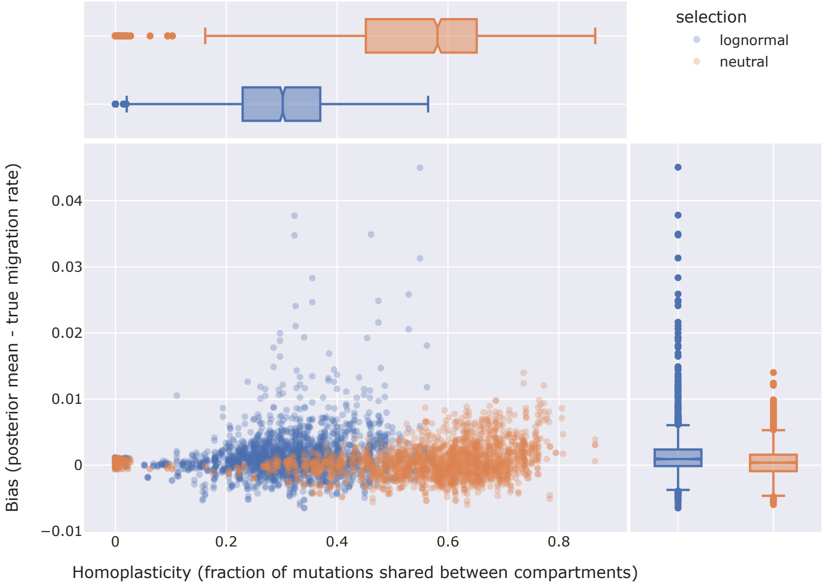

Fig. S15: Homoplasticity vs. Bias in migration rate estimates with relaxed clock model on simulations with a constant population size of 100 virions per compartment and a mutation rate of 2.16×10^{-5} mutation bp^{-1} generation^{-1}.

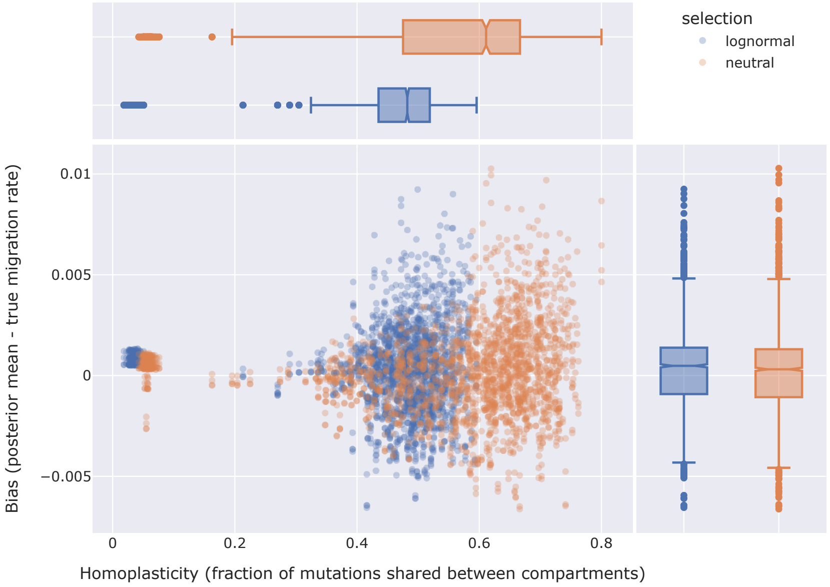

Fig. S16: Homoplasticity vs. Bias in migration rate estimates with relaxed clock model on simulations with a constant population size of 100 virions per compartment and a mutation rate of 2.16×10^{-4} mutation bp^{-1} generation^{-1}.

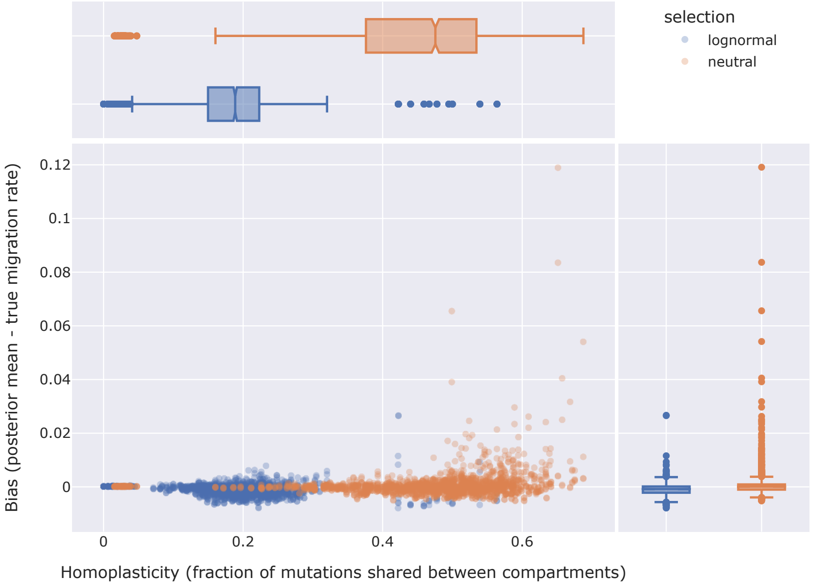

Fig. S17: Homoplasticity vs. Bias in migration rate estimates with relaxed clock model on simulations with a constant population size of 1000 virions per compartment and a mutation rate of 2.16×10^{-5} mutation bp^{-1} generation^{-1}.

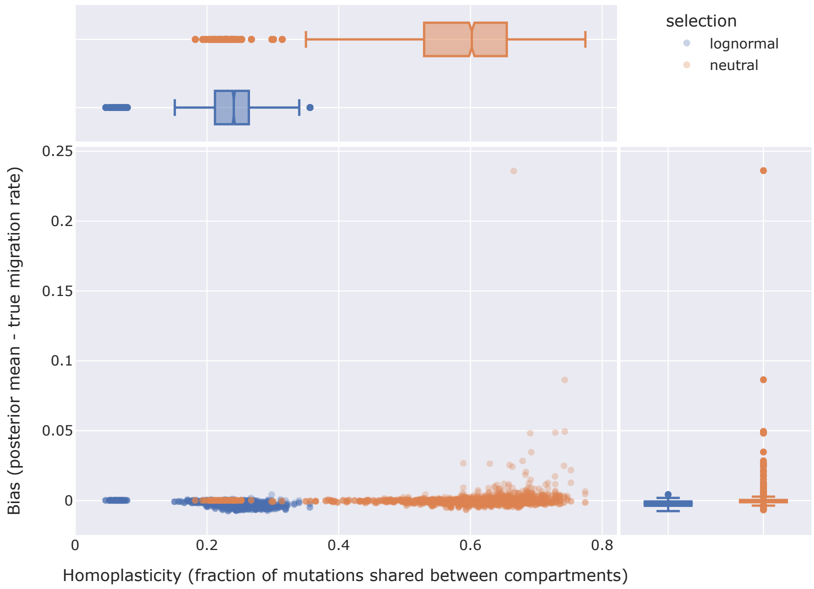

Fig. S18: Homoplasticity vs. Bias in migration rate estimates with relaxed clock model on simulations with a constant population size of 1000 virions per compartment and a mutation rate of 2.16×10^{-4} mutation bp^{-1} generation^{-1}.

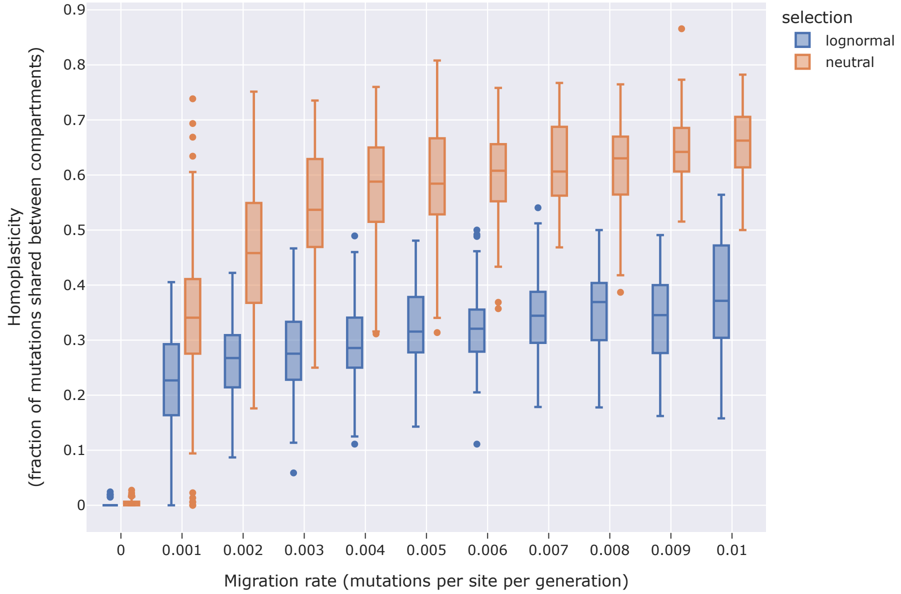

Fig. S19: Migration rate vs. homoplasticity in migration rate estimates with relaxed clock model on simulations with a constant population size of 100 virions per compartment and a mutation rate of 2.16×10^{-5} mutation bp^{-1} generation^{-1}.

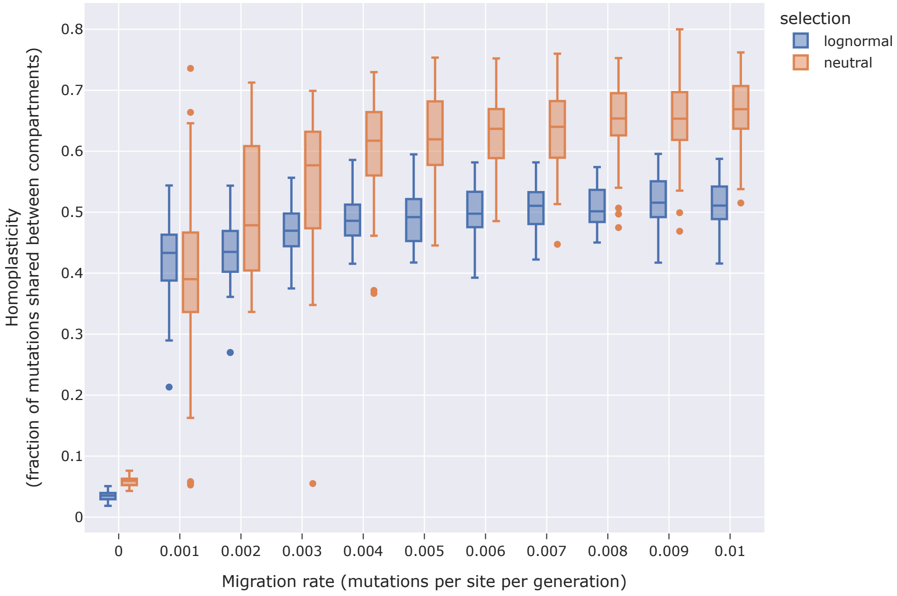

Fig. S20: Migration rate vs. homoplasticity in migration rate estimates with relaxed clock model on simulations with a constant population size of 100 virions per compartment and a mutation rate of 2.16×10^{-4} mutation bp^{-1} generation^{-1}.

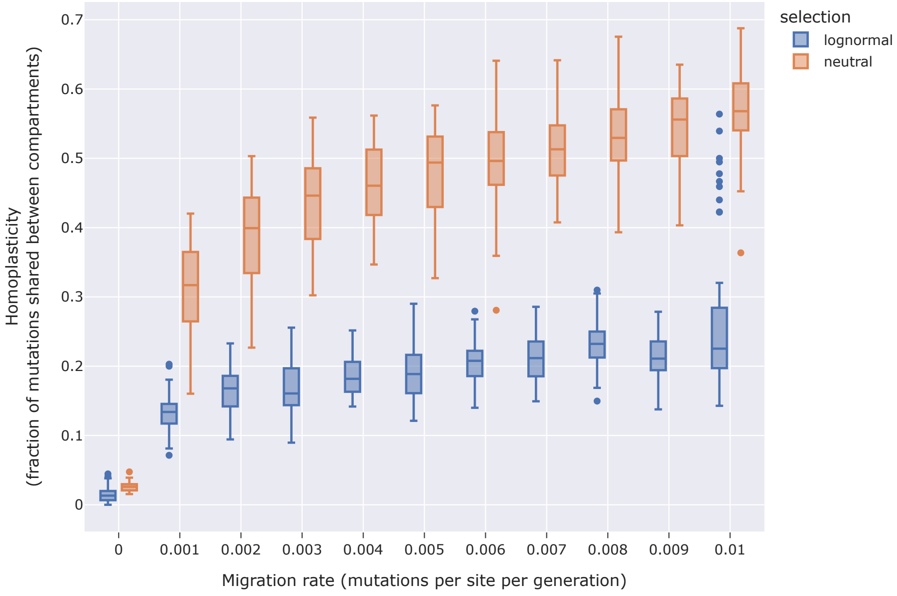

Fig. S21: Migration rate vs. homoplasticity in migration rate estimates with relaxed clock model on simulations with a constant population size of 1000 virions per compartment and a mutation rate of 2.16×10^{-5} mutation bp^{-1} generation^{-1}.

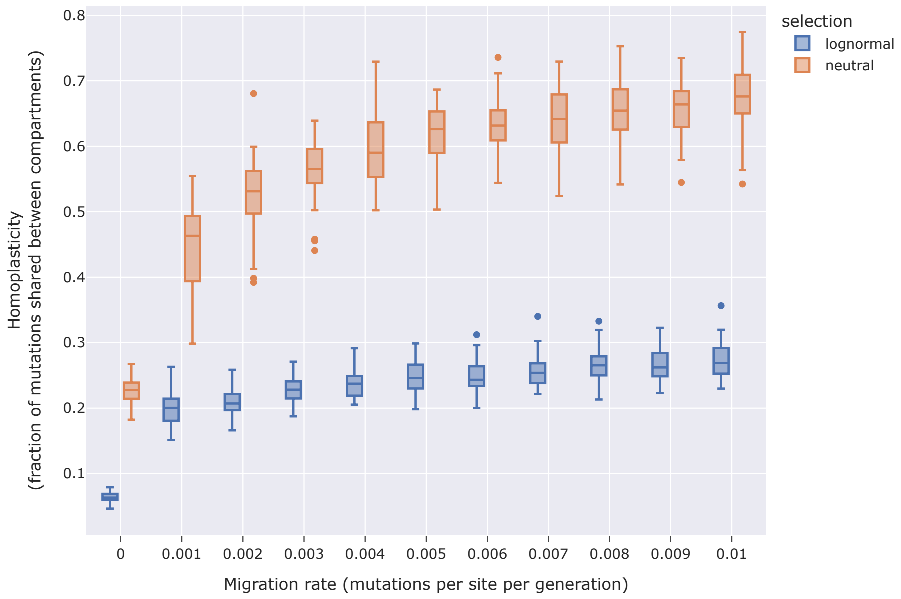

Fig. S22: Migration rate vs. homoplasticity in migration rate estimates with relaxed clock model on simulations with a constant population size of 1000 virions per compartment and a mutation rate of 2.16×10^{-4} mutation bp^{-1} generation^{-1}.

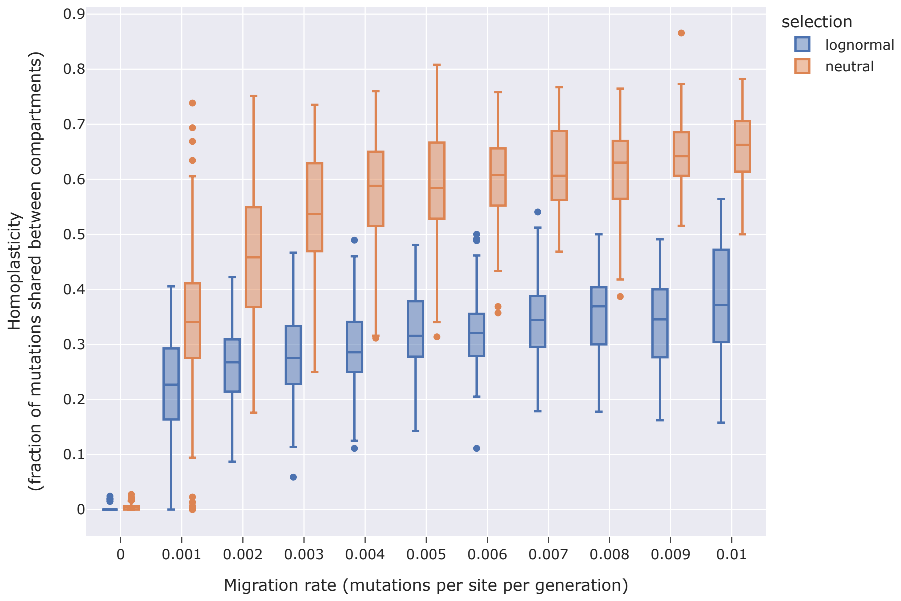

Fig. S23: Migration rate vs. homoplasticity in migration rate estimates with relaxed clock model on simulations with a constant population size of 100 virions per compartment and a mutation rate of 2.16×10^{-5} mutation bp^{-1} generation^{-1}.

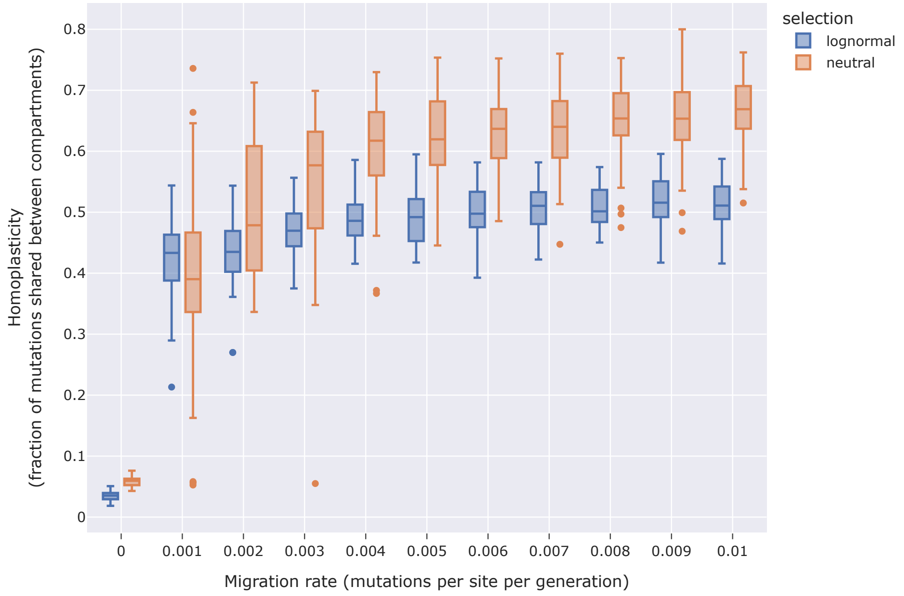

Fig. S24: Migration rate vs. homoplasticity in migration rate estimates with relaxed clock model on simulations with a constant population size of 100 virions per compartment and a mutation rate of 2.16×10^{-4} mutation bp^{-1} generation^{-1}.

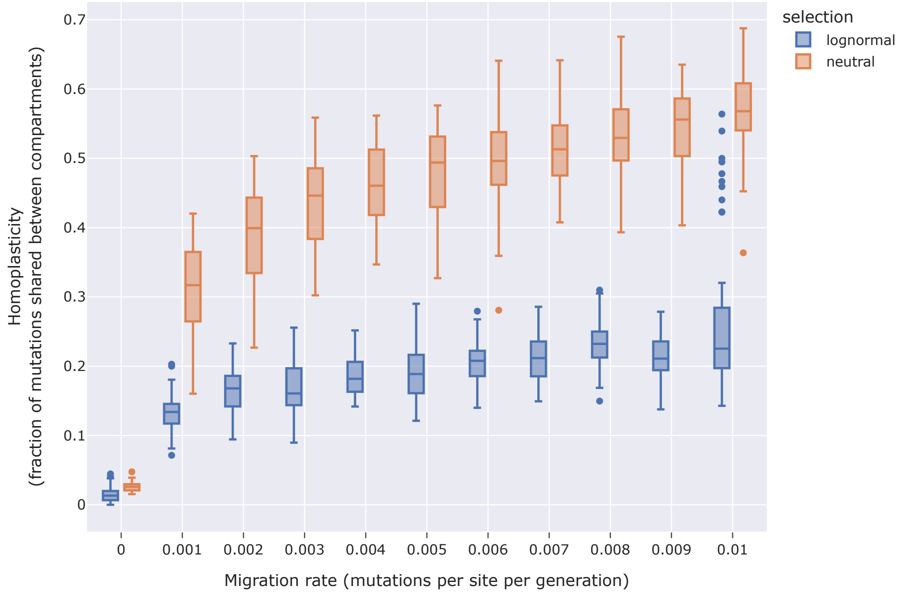

Fig. S25: Migration rate vs. homoplasticity in migration rate estimates with relaxed clock model on simulations with a constant population size of 1000 virions per compartment and a mutation rate of 2.16×10^{-5} mutation bp^{-1} generation^{-1}.

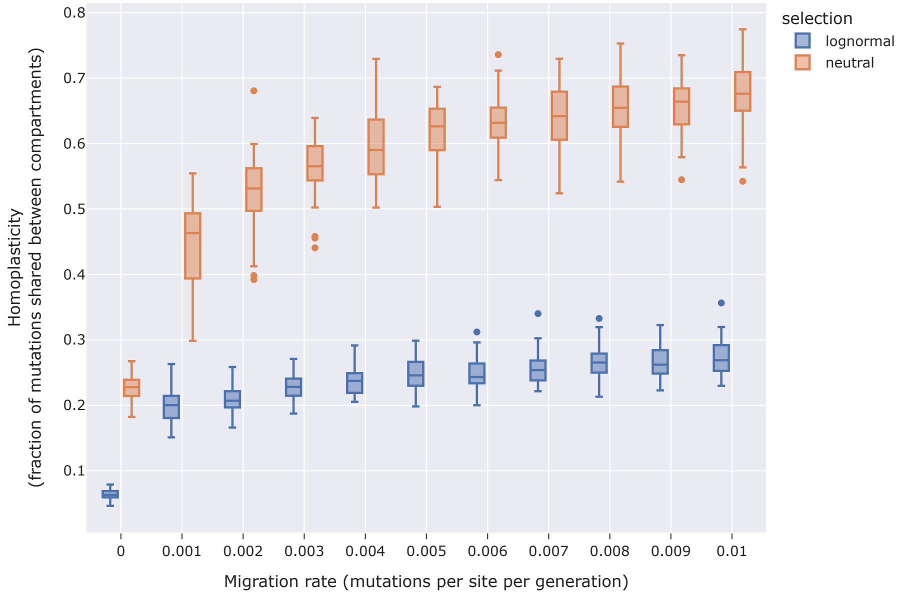

Fig. S26: Migration rate vs. homoplasticity in migration rate estimates with relaxed clock model on simulations with a constant population size of 1000 virions per compartment and a mutation rate of 2.16×10^{-4} mutation bp^{-1} generation^{-1}.

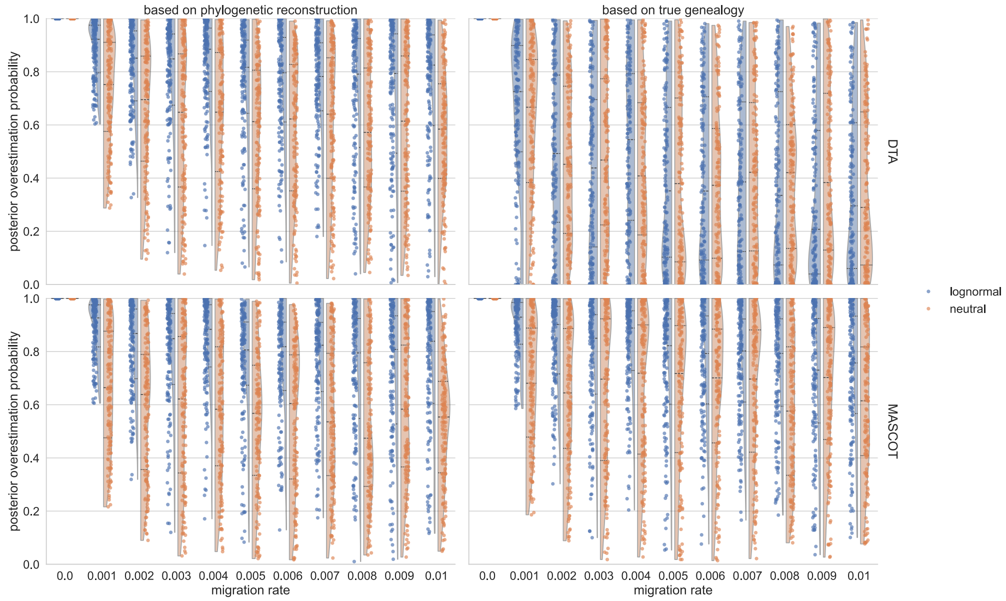

Fig. S27: **Posterior Overestimation Probability with Relaxed Clock Model.** Posterior probability of migration rate overestimation. For each posterior sample, we calculated the posterior probability of overestimation after an initial burn-in of 10% of the samples. For each migration rate, 50 independent simulations were conducted with a constant population of 100 virions per compartment, a mutation rate of 2.16×10^{-5} mutation bp^{-1} generation^{-1}, and a total of 1,000 generations. All sequences were sampled and analyzed in BEAST2 with a relaxed clock and fixed effective population size. The left figures illustrate the inference while sampling trees, while the right figures display the migration rate estimates based on the true – simulated – genealogy.

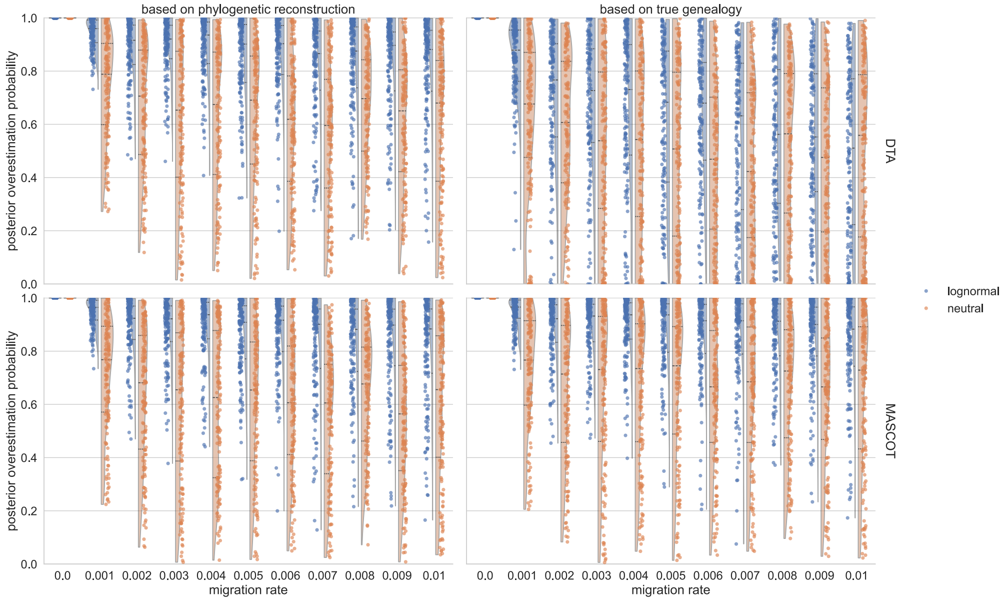

Fig. S28: **Posterior Overestimation Probability with Relaxed Clock Model.** Posterior probability of migration rate overestimation. For each posterior sample, we calculated the posterior probability of overestimation after an initial burn-in of 10% of the samples. For each migration rate, 50 independent simulations were conducted with a constant population of 100 virions per compartment, a mutation rate of 2.16×10^{-4} mutation bp^{-1} generation^{-1}, and a total of 1,000 generations. All sequences were sampled and analyzed in BEAST2 with a relaxed clock and fixed effective population size. The left figures illustrate the inference while sampling trees, while the right figures display the migration rate estimates based on the true – simulated – genealogy.

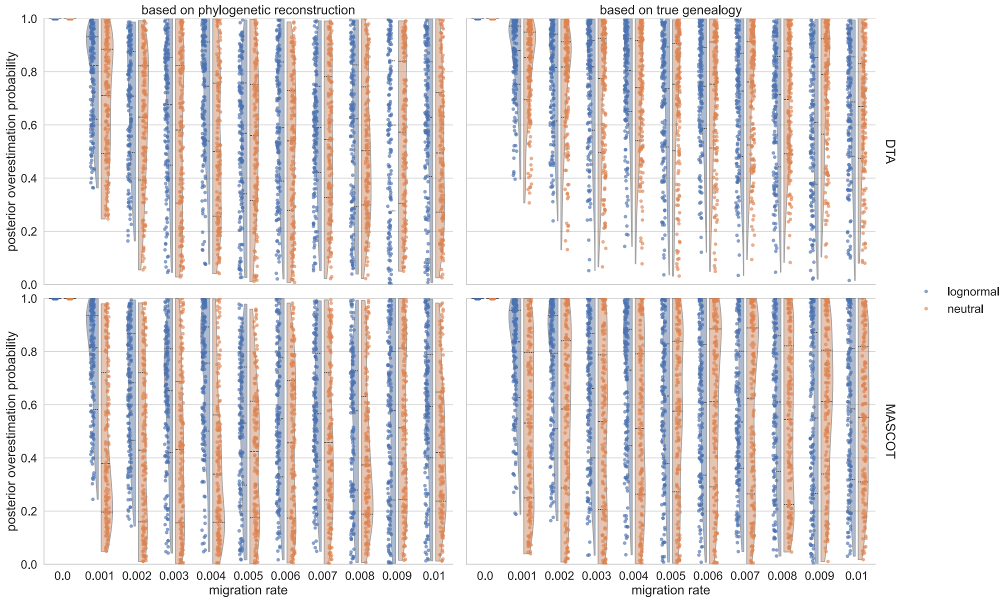

Fig. S29: **Posterior Overestimation Probability with Relaxed Clock Model.** Posterior probability of migration rate overestimation. For each posterior sample, we calculated the posterior probability of overestimation after an initial burn-in of 10% of the samples. For each migration rate, 50 independent simulations were conducted with a constant population of 100 virions per compartment, a mutation rate of 2.16×10^{-5} mutation bp^{-1} generation^{-1}, and a total of 1,000 generations. All sequences were sampled and analyzed in BEAST2 with a strict clock and fixed effective population size. The left figures illustrate the inference while sampling trees, while the right figures display the migration rate estimates based on the true – simulated – genealogy.

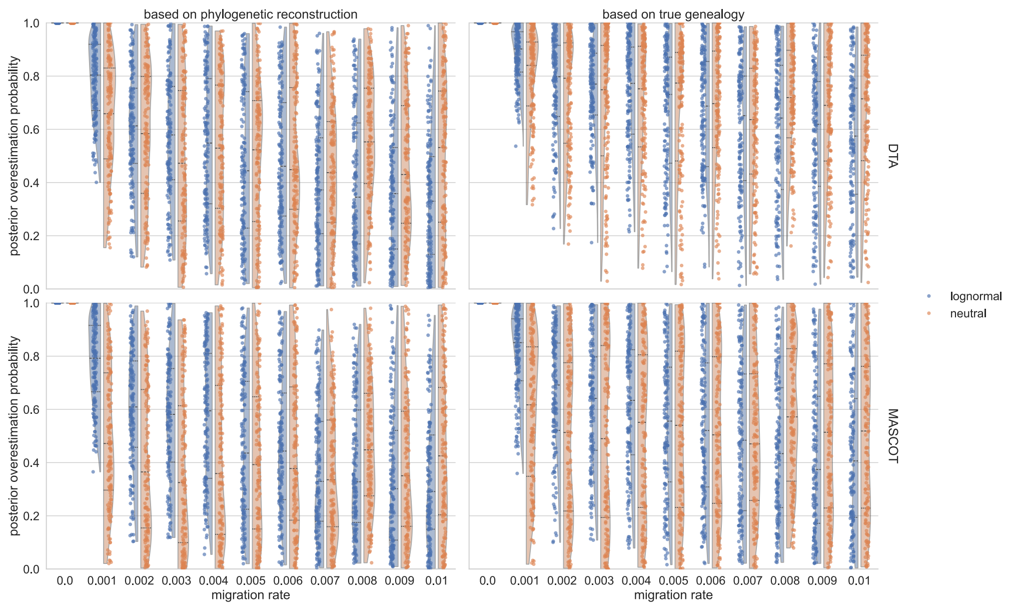

Fig. S30: **Posterior Overestimation Probability with Relaxed Clock Model.** Posterior probability of migration rate overestimation. For each posterior sample, we calculated the posterior probability of overestimation after an initial burn-in of 10% of the samples. For each migration rate, 50 independent simulations were conducted with a constant population of 100 virions per compartment, a mutation rate of 2.16×10^{-4} mutation bp^{-1} generation^{-1}, and a total of 1,000 generations. All sequences were sampled and analyzed in BEAST2 with a strict clock and fixed effective population size. The left figures illustrate the inference while sampling trees, while the right figures display the migration rate estimates based on the true – simulated – genealogy.

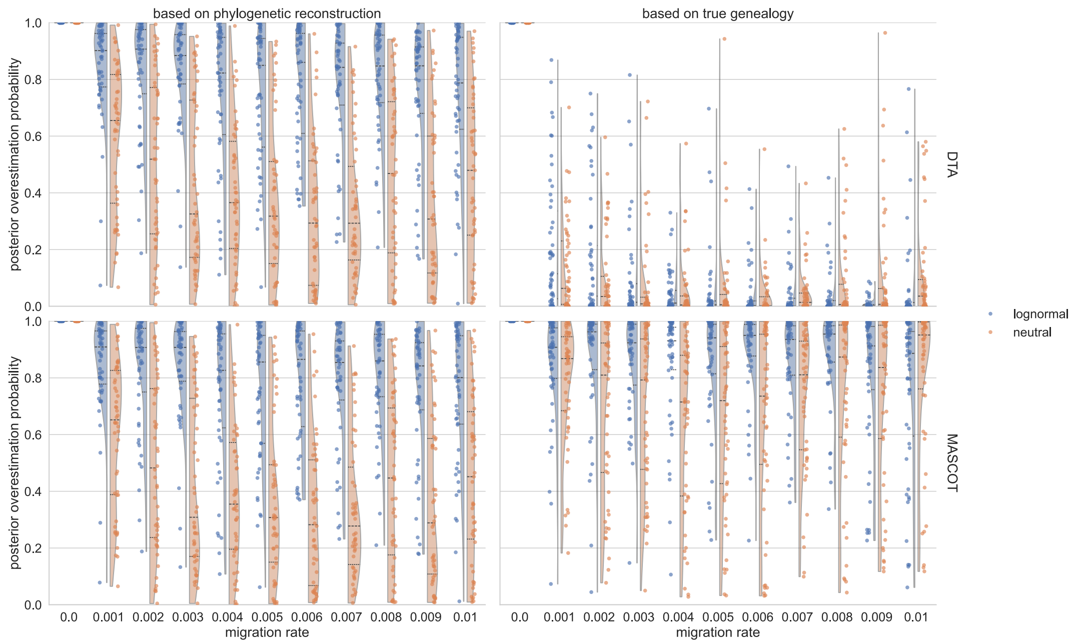

Fig. S31: **Posterior Overestimation Probability with Relaxed Clock Model.** Posterior probability of migration rate overestimation. For each posterior sample, we calculated the posterior probability of overestimation after an initial burn-in of 10% of the samples. For each migration rate, 50 independent simulations were conducted with a constant population of 1000 virions per compartment, a mutation rate of 2.16×10^{-5} mutation bp^{-1} generation^{-1}, and a total of 1,000 generations. All sequences were sampled and analyzed in BEAST2 with a relaxed clock and fixed effective population size. The left figures illustrate the inference while sampling trees, while the right figures display the migration rate estimates based on the true – simulated – genealogy.

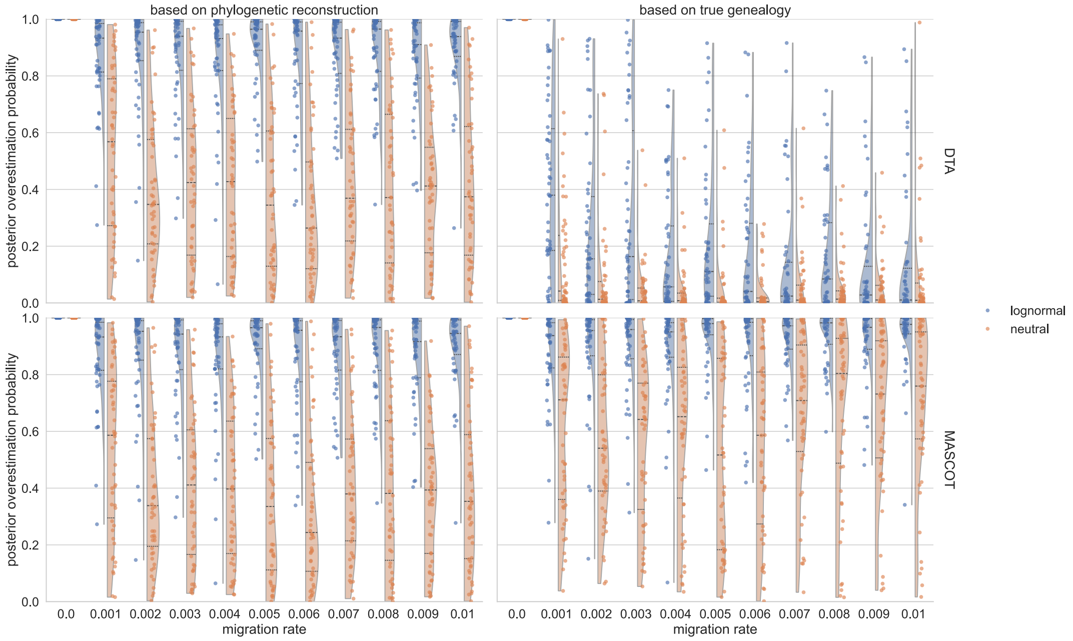

Fig. S32: **Posterior Overestimation Probability with Relaxed Clock Model.** Posterior probability of migration rate overestimation. For each posterior sample, we calculated the posterior probability of overestimation after an initial burn-in of 10% of the samples. For each migration rate, 50 independent simulations were conducted with a constant population of 1000 virions per compartment, a mutation rate of 2.16×10^{-4} mutation bp^{-1} generation^{-1}, and a total of 1,000 generations. All sequences were sampled and analyzed in BEAST2 with a relaxed clock and fixed effective population size. The left figures illustrate the inference while sampling trees, while the right figures display the migration rate estimates based on the true – simulated – genealogy.

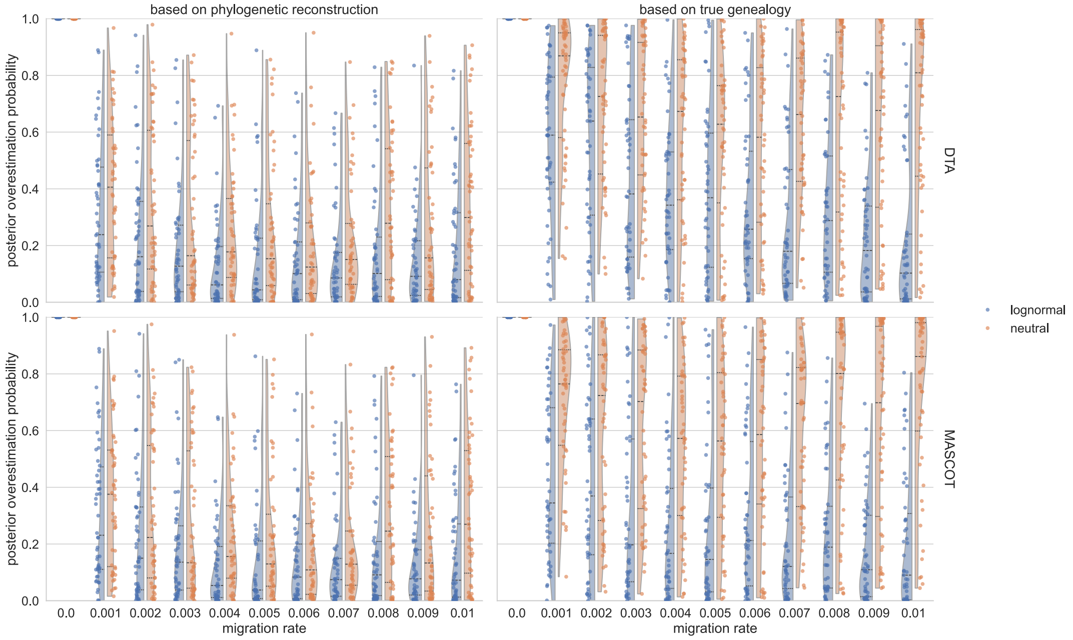

Fig. S33: **Posterior Overestimation Probability with Relaxed Clock Model.** Posterior probability of migration rate overestimation. For each posterior sample, we calculated the posterior probability of overestimation after an initial burn-in of 10% of the samples. For each migration rate, 50 independent simulations were conducted with a constant population of 1000 virions per compartment, a mutation rate of 2.16×10^{-5} mutation bp^{-1} generation^{-1}, and a total of 1,000 generations. All sequences were sampled and analyzed in BEAST2 with a strict clock and fixed effective population size. The left figures illustrate the inference while sampling trees, while the right figures display the migration rate estimates based on the true – simulated – genealogy.

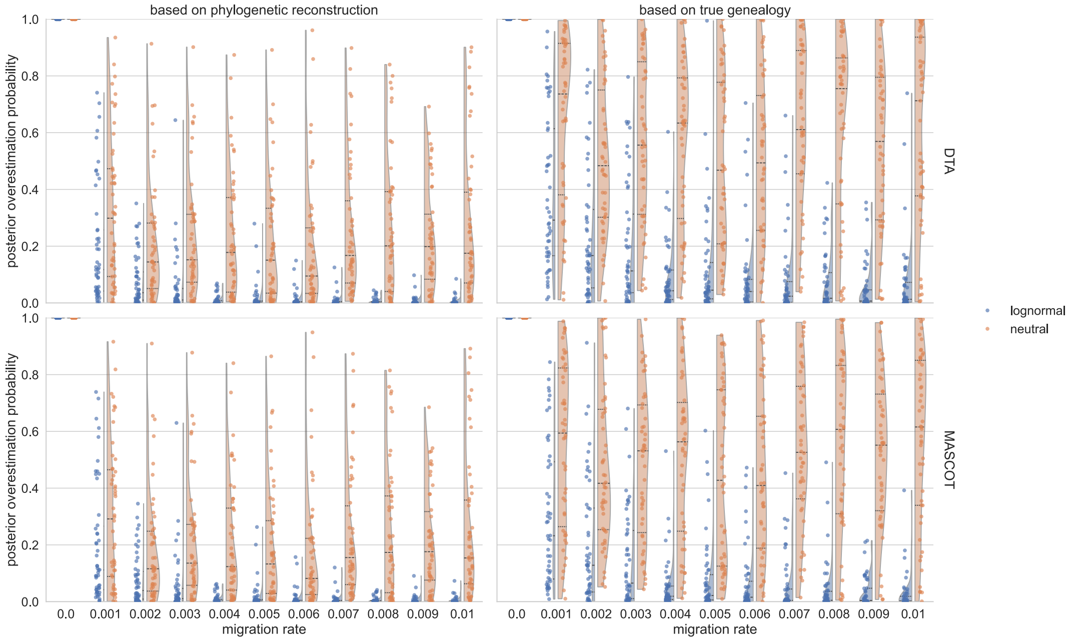

Fig. S34: **Posterior Overestimation Probability with Relaxed Clock Model.** Posterior probability of migration rate overestimation. For each posterior sample, we calculated the posterior probability of overestimation after an initial burn-in of 10% of the samples. For each migration rate, 50 independent simulations were conducted with a constant population of 1000 virions per compartment, a mutation rate of 2.16×10^{-4} mutation bp^{-1} generation^{-1}, and a total of 1,000 generations. All sequences were sampled and analyzed in BEAST2 with a strict clock and fixed effective population size. The left figures illustrate the inference while sampling trees, while the right figures display the migration rate estimates based on the true – simulated – genealogy.

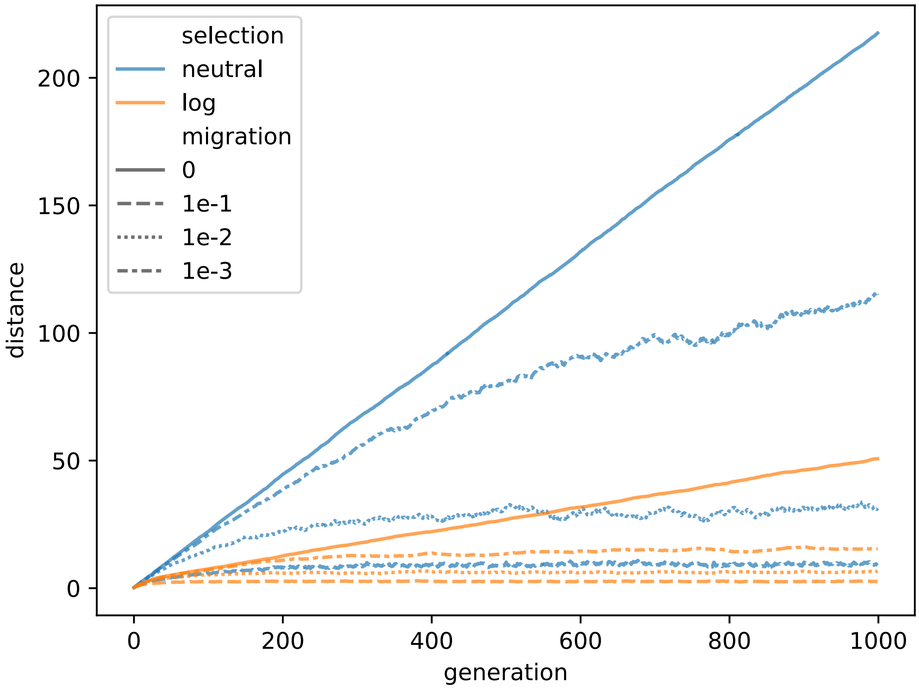

Fig. S35: **Genetic distance between the two compartments.** Average distance between populations in two compartments in 100 simulations with mutation rate 2.16×10^{-5} mutation bp^{-1} generation^{-1}with and without selection for selected migration rates (0, 0.1, 0.01, 0.001). The distance is calculated by summing the absolute differences in nucleotide frequencies between compartments.

				selection	neutral											lognormal										
				migration_rate	0e+00	1e-03	2e-03	3e-03	4e-03	5e-03	6e-03	7e-03	8e-03	9e-03	1e-02	0e+00	1e-03	2e-03	3e-03	4e-03	5e-03	6e-03	7e-03	8e-03	9e-03	1e-02
method	tree	population size	n_samples	mutation rate per bp per generation																						
lemey	true	50	100	2.16e-5	6.34e-07	2.89e-06	1.10e-06	8.83e-06	3.25e-05	3.48e-05	1.06e-05	2.20e-05	3.29e-04	4.45e-04	6.66e-04	9.02e-07	2.15e-06	3.12e-06	8.05e-06	1.51e-05	7.89e-06	1.48e-05	1.13e-05	2.31e-05	6.15e-05	5.19e-04
				2.16e-4	8.85e-07	1.83e-06	3.71e-06	5.79e-06	8.17e-06	2.67e-05	1.61e-05	2.20e-05	7.33e-05	2.58e-05	9.28e-05	2.01e-06	7.95e-06	9.71e-06	2.08e-05	1.24e-04	1.72e-05	3.34e-05	2.22e-05	4.44e-05	1.19e-03	4.70e-05
		100	100	2.16e-5	5.02e-07	9.61e-07	1.34e-06	2.36e-06	3.10e-06	5.78e-06	7.56e-06	7.89e-06	1.07e-05	1.54e-05	2.11e-05	5.07e-07	1.01e-06	1.63e-06	1.94e-06	2.92e-06	5.79e-06	7.37e-06	1.07e-05	1.87e-05	2.27e-05	2.34e-05
				2.16e-4	8.34e-07	1.71e-06	2.12e-06	6.10e-06	6.05e-06	5.36e-06	7.26e-06	8.83e-06	1.33e-05	1.52e-05	2.72e-05	1.70e-06	5.25e-06	5.90e-06	6.66e-06	1.03e-05	1.39e-05	1.26e-05	1.06e-05	1.50e-05	1.68e-05	1.64e-05
		200	1000	2.16e-5	1.70e-08	1.85e-07	8.92e-07	2.20e-06	4.43e-06	6.18e-06	1.00e-05	1.06e-05	1.52e-05	1.92e-05	4.09e-05	2.06e-08	1.79e-07	8.72e-07	2.04e-06	3.80e-06	5.80e-06	7.99e-06	1.29e-05	1.60e-05	2.26e-05	2.81e-05
				2.16e-4	1.84e-08	2.03e-07	1.03e-06	2.10e-06	4.20e-06	6.90e-06	1.01e-05	1.13e-05	1.48e-05	1.87e-05	5.34e-04	7.10e-08	3.56e-07	6.83e-07	2.04e-06	2.59e-06	3.09e-06	5.98e-06	8.73e-06	9.61e-06	1.62e-05	1.87e-05
			100	2.16e-5	1.02e-06	2.38e-06	3.33e-06	8.06e-06	8.67e-06	9.75e-06	9.63e-06	1.62e-05	9.33e-06	1.90e-05	1.19e-05	1.18e-06	3.91e-06	5.02e-06	5.30e-06	9.93e-06	9.14e-06	1.00e-05	1.24e-05	1.75e-05	1.89e-05	1.74e-05
				2.16e-4	9.86e-07	2.78e-06	3.41e-06	6.22e-06	8.34e-06	9.74e-06	9.77e-06	1.10e-05	1.34e-05	1.44e-05	1.94e-05	1.82e-06	6.67e-06	6.19e-06	1.14e-05	1.55e-05	1.45e-05	1.72e-05	1.56e-05	1.75e-05	1.62e-05	1.67e-05
	reconstructed	50	100	2.16e-5	1.70e-06	8.60e-06	1.06e-05	3.97e-05	5.11e-05	7.33e-05	4.78e-05	1.08e-04	3.72e-04	2.94e-04	3.35e-04	6.10e-06	5.49e-05	4.69e-05	4.58e-04	2.25e-03	1.46e-04	1.47e-03	8.00e-04	7.03e-04	2.42e-03	4.93e-03
				2.16e-4	1.60e-06	5.33e-06	1.10e-05	1.94e-05	3.25e-05	4.30e-05	1.01e-04	8.85e-05	1.15e-04	4.29e-04	2.72e-04	5.12e-06	4.36e-05	4.57e-05	1.27e-04	1.25e-04	1.55e-04	2.05e-04	1.72e-04	2.41e-04	2.71e-04	3.31e-04
		100	100	2.16e-5	1.05e-06	3.43e-06	4.98e-06	1.25e-05	1.90e-05	1.36e-05	1.53e-05	3.24e-05	3.96e-05	5.05e-05	4.32e-05	3.44e-06	1.55e-05	1.71e-05	4.41e-05	8.25e-05	3.17e-05	1.32e-04	8.13e-05	1.72e-04	5.90e-04	1.28e-03
				2.16e-4	9.87e-07	3.34e-06	3.99e-06	8.66e-06	9.24e-06	1.74e-05	1.68e-05	1.44e-05	2.33e-05	2.34e-05	4.36e-05	3.26e-06	2.30e-05	2.19e-05	4.07e-05	5.60e-05	5.23e-05	8.23e-05	7.29e-05	8.58e-05	9.49e-05	8.49e-05
		200	1000	2.16e-5	4.24e-08	1.53e-07	4.97e-07	7.36e-07	1.07e-06	1.95e-06	2.45e-06	2.45e-06	5.09e-06	6.58e-06	8.34e-06	1.98e-07	1.55e-06	4.80e-06	7.29e-06	7.26e-06	1.22e-05	1.79e-05	2.17e-05	3.20e-05	2.79e-05	1.45e-04
				2.16e-4	4.68e-08	1.60e-07	2.73e-07	5.91e-07	8.93e-07	1.84e-06	3.02e-06	2.68e-06	4.67e-06	3.31e-06	6.78e-06	5.45e-07	2.94e-06	6.32e-06	1.10e-05	9.56e-06	2.10e-05	2.44e-05	2.61e-05	3.43e-05	3.72e-05	4.51e-05
			100	2.16e-5	7.59e-07	1.87e-06	2.49e-06	6.13e-06	8.08e-06	7.74e-06	7.97e-06	1.27e-05	1.60e-05	1.96e-05	1.00e-05	2.23e-06	8.44e-06	8.04e-06	1.76e-05	4.36e-05	1.52e-05	5.26e-05	3.47e-05	5.50e-05	1.26e-04	2.48e-04
				2.16e-4	7.17e-07	1.89e-06	2.28e-06	5.11e-06	5.30e-06	7.41e-06	7.31e-06	8.17e-06	9.87e-06	1.13e-05	1.75e-05	2.36e-06	1.35e-05	1.27e-05	2.70e-05	3.55e-05	3.28e-05	4.05e-05	4.01e-05	4.52e-05	5.06e-05	4.83e-05
mascot	true	50	100	2.16e-5	4.10e-06	2.21e-05	3.11e-05	1.64e-04	3.16e-04	3.10e-04	1.52e-03	6.46e-04	1.05e-03	4.71e-03	3.36e-03	1.16e-05	4.02e-05	5.15e-05	2.34e-04	4.14e-04	2.67e-04	2.92e-03	1.29e-03	6.93e-04	9.53e-04	2.20e-03
				2.16e-4	2.04e-05	1.88e-05	6.54e-05	2.25e-04	1.17e-04	3.44e-04	7.25e-04	1.73e-04	4.58e-04	8.39e-04	9.61e-04	7.45e-06	4.86e-05	6.82e-05	1.08e-04	1.88e-04	2.61e-04	3.20e-04	3.04e-04	5.48e-04	4.58e-04	1.40e-03
		100	100	2.16e-5	1.05e-04	3.87e-05	1.52e-05	7.15e-05	1.50e-04	1.49e-04	1.23e-04	1.00e-04	5.37e-05	1.20e-04	6.87e-05	7.37e-06	1.33e-05	4.16e-05	7.64e-05	5.21e-05	4.05e-05	8.39e-05	5.27e-05	1.51e-04	1.73e-04	2.95e-04
				2.16e-4	1.11e-04	4.04e-05	5.79e-05	4.70e-05	1.54e-04	5.19e-05	5.22e-05	2.35e-04	5.04e-05	4.28e-05	1.95e-04	4.07e-06	2.51e-05	2.61e-05	4.36e-05	8.21e-05	5.88e-05	9.03e-05	1.23e-04	1.25e-04	1.35e-04	1.93e-04
		200	1000	2.16e-5	7.63e-08	9.08e-07	2.46e-06	3.10e-06	3.86e-06	8.79e-06	1.49e-05	2.00e-05	8.17e-05	1.84e-04	1.45e-03	2.23e-07	1.37e-06	4.22e-06	8.73e-06	1.03e-05	1.96e-05	2.53e-05	2.47e-05	3.74e-05	3.09e-05	4.46e-05
				2.16e-4	7.62e-08	3.05e-07	1.50e-06	1.30e-06	8.59e-06	4.40e-06	8.63e-06	2.59e-05	4.68e-05	4.23e-05	5.89e-04	5.66e-07	3.37e-06	7.49e-06	1.33e-05	1.28e-05	3.11e-05	3.73e-05	4.52e-05	6.41e-05	6.75e-05	8.91e-05
			100	2.16e-5	5.06e-07	8.31e-07	1.56e-06	4.04e-06	4.44e-06	4.67e-06	4.03e-06	7.40e-06	5.16e-06	8.24e-06	4.31e-06	2.03e-06	4.15e-06	4.92e-06	4.68e-06	8.16e-06	7.17e-06	7.33e-06	8.00e-06	1.24e-05	1.30e-05	1.42e-05
				2.16e-4	5.71e-07	1.52e-06	1.85e-06	4.59e-06	5.16e-06	6.47e-06	6.46e-06	7.97e-06	8.32e-06	8.52e-06	1.37e-05	2.36e-06	1.17e-05	1.25e-05	2.29e-05	3.26e-05	3.11e-05	3.84e-05	3.90e-05	4.30e-05	4.64e-05	4.97e-05
	reconstructed	50	100	2.16e-5	8.65e-07	6.19e-06	5.10e-06	4.48e-05	9.65e-05	2.02e-05	3.75e-05	7.73e-05	6.16e-04	6.46e-04	4.99e-04	5.96e-06	1.48e-04	5.35e-05	1.19e-03	2.81e-03	9.31e-05	2.62e-03	5.17e-03	3.90e-03	1.43e-02	2.90e-02
				2.16e-4	8.82e-07	5.37e-06	8.60e-06	1.59e-05	2.24e-05	4.69e-05	9.65e-05	2.09e-05	9.62e-05	6.94e-05	1.94e-04	4.80e-06	5.35e-05	4.72e-05	1.37e-04	1.58e-04	1.18e-04	1.75e-04	1.66e-04	3.25e-04	3.68e-04	6.34e-04
		100	100	2.16e-5	6.81e-07	2.30e-06	4.31e-06	9.65e-06	8.42e-06	1.58e-05	1.16e-05	2.06e-05	3.34e-05	4.11e-05	1.89e-05	3.46e-06	1.67e-05	1.65e-05	4.54e-05	8.80e-05	2.45e-05	1.56e-04	2.21e-04	2.47e-04	5.05e-03	2.31e-03
				2.16e-4	7.05e-07	2.60e-06	3.26e-06	6.85e-06	9.32e-06	1.54e-05	1.34e-05	1.22e-05	1.80e-05	2.27e-05	4.54e-05	3.26e-06	2.27e-05	2.37e-05	3.56e-05	6.37e-05	4.75e-05	7.89e-05	7.57e-05	8.16e-05	9.10e-05	1.26e-04
		200	1000	2.16e-5	4.29e-08	1.47e-07	4.73e-07	7.06e-07	1.05e-06	1.91e-06	2.47e-06	2.47e-06	4.95e-06	6.54e-06	8.14e-06	1.98e-07	1.58e-06	4.89e-06	7.62e-06	7.60e-06	1.26e-05	1.82e-05	2.17e-05	3.23e-05	2.78e-05	1.86e-04
				2.16e-4	4.74e-08	1.52e-07	2.71e-07	5.69e-07	8.63e-07	1.84e-06	3.00e-06	2.53e-06	4.58e-06	3.32e-06	6.52e-06	5.42e-07	2.95e-06	6.35e-06	1.11e-05	9.62e-06	2.12e-05	2.46e-05	2.64e-05	3.49e-05	3.78e-05	4.65e-05
			100	2.16e-5	5.69e-07	1.35e-06	2.04e-06	5.54e-06	7.09e-06	6.34e-06	6.83e-06	1.11e-05	1.42e-05	1.78e-05	8.49e-06	2.24e-06	8.36e-06	8.14e-06	1.68e-05	4.21e-05	1.52e-05	4.83e-05	3.31e-05	5.06e-05	1.09e-04	2.08e-04
				2.16e-4	6.02e-07	1.66e-06	1.98e-06	4.85e-06	5.04e-06	6.97e-06	6.85e-06	8.07e-06	9.25e-06	1.04e-05	1.63e-05	2.41e-06	1.37e-05	1.28e-05	2.72e-05	3.62e-05	3.31e-05	4.17e-05	4.08e-05	4.63e-05	5.22e-05	5.10e-05

Table S4. Mean Squared Error (MSE) of Migration Rate Estimates in Relaxed Clock Model across all evaluated scenarios, based on a total of 50 simulations with a constant number of virions in each compartment.

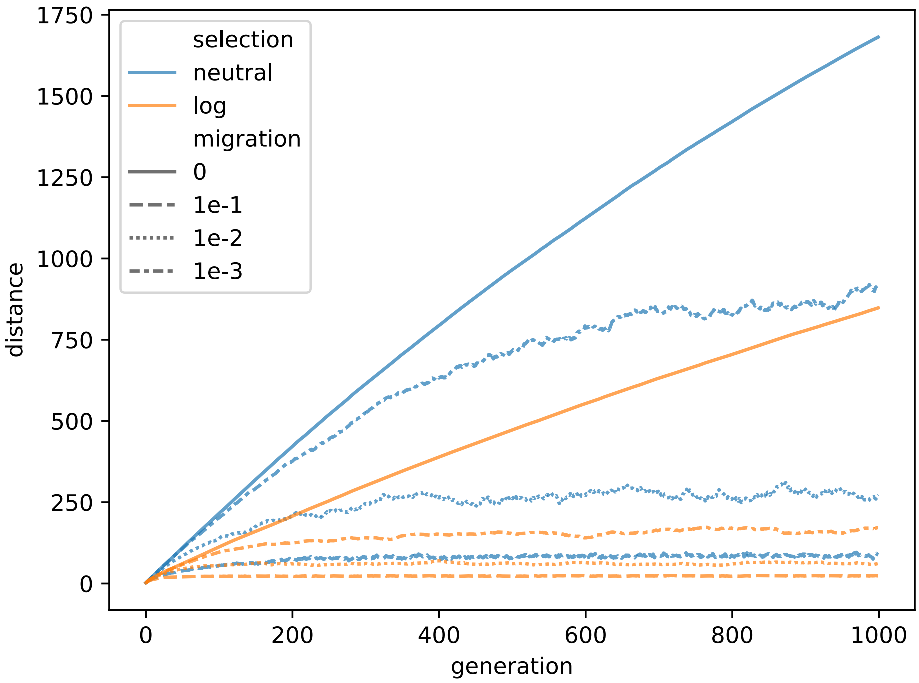

Fig. S36: **Genetic distance between the two compartments.** Average distance between populations in two compartments in 100 simulations with mutation rate 2.16×10^{-4} mutation bp^{-1} generation^{-1}with and without selection for selected migration rates (0, 0.1, 0.01, 0.001). The distance is calculated by summing the absolute differences in nucleotide frequencies between compartments.
